# The *Plasmodium* PHIST and RESA-Like Protein Families of Human and Rodent Malaria Parasites

**DOI:** 10.1371/journal.pone.0152510

**Published:** 2016-03-29

**Authors:** Cristina K. Moreira, Bernina Naissant, Alida Coppi, Brandy L. Bennett, Elena Aime, Blandine Franke-Fayard, Chris J. Janse, Isabelle Coppens, Photini Sinnis, Thomas J. Templeton

**Affiliations:** 1 Department of Microbiology and Immunology, Weill Cornell Medical College, New York, NY 10021, United States of America; 2 Department of Medical Parasitology, NYU School of Medicine, New York, NY, 10010, United States of America; 3 Leiden Malaria Research Group, Department of Parasitology, Leiden University Medical Centre, Leiden, The Netherlands; 4 Department of Molecular Microbiology and Immunology, Johns Hopkins Bloomberg School of Public Health, Baltimore, MD, 21205, United States of America; 5 Department of Protozoology, Institute of Tropical Medicine (NEKKEN), Nagasaki 852-8523, Japan; Institut national de la santé et de la recherche médicale - Institut Cochin, FRANCE

## Abstract

The *phist* gene family has members identified across the *Plasmodium* genus, defined by the presence of a domain of roughly 150 amino acids having conserved aromatic residues and an all alpha-helical structure. The family is highly amplified in *P*. *falciparum*, with 65 predicted genes in the genome of the 3D7 isolate. In contrast, in the rodent malaria parasite *P*. *berghei* 3 genes are identified, one of which is an apparent pseudogene. Transcripts of the *P*. *berghei phist* genes are predominant in schizonts, whereas in *P*. *falciparum* transcript profiles span different asexual blood stages and gametocytes. We pursued targeted disruption of *P*. *berghei phist* genes in order to characterize a simplistic model for the expanded *phist* gene repertoire in *P*. *falciparum*. Unsuccessful attempts to disrupt *P*. *berghei PBANKA_114540* suggest that this *phist* gene is essential, while knockout of *phist PBANKA_122900* shows an apparent normal progression and non-essential function throughout the life cycle. Epitope-tagging of *P*. *falciparum* and *P*. *berghei phist* genes confirmed protein export to the erythrocyte cytoplasm and localization with a punctate pattern. Three *P*. *berghei* PEXEL/HT-positive exported proteins exhibit at least partial co-localization, in support of a common vesicular compartment in the cytoplasm of erythrocytes infected with rodent malaria parasites.

## Introduction

The subtelomeric regions of chromosomes of the human malaria parasite, *Plasmodium falciparum*, are compartmentalized such that highly amplified and hypervariable genes are relegated within discrete gene neighborhoods; the majority, but not all of which are sub-telomeric [1,2]. These genes predominantly encode proteins that are exported to the cytoplasm and membrane of the infected erythrocyte, and the most telomere-proximal regions are composed of highly variable genes; namely, the *var*, *rifin* and *stevor* genes, which encode erythrocyte surface membrane proteins that are exposed to host antibody-mediated immune pressure. Instability of the subtelomeric regions is thought to facilitate rapid changes in the repertoires of these gene families via poorly understood processes of recombination, gene conversion and DNA repair, which are selected for via a spectrum of host immune and other adaptive pressures that are encountered by the parasite ([3–5]; reviewed in [6]). Thus the chromosome ends might be considered to be incubators for rapid evolution, whereas the more central regions of the chromosomes are protected from instability in order to safeguard “housekeeping” metabolic functions of the parasite [7]. In cultured parasites the lability of the chromosome ends frequently manifests as subtelomeric breakage and the loss of blocks of genes that are not essential for *in vitro* growth [8, 9]. Adjacent and internal to the *var*, *rifin* and *stevor* genes are neighborhoods composed of additional highly amplified gene families, the products of which are also predicted to be transported to the erythrocyte cytoplasm but are thought to not be exposed to host immune pressure. These families include the 20 plus member family of FIKK threonine/serine kinases [10]; lysophospholipases; fatty acyl synthetases [11] and, the subject of the present study, the highly amplified *phist* (*P**lasmodium* helical interspersed subtelomeric) and *resa-like* genes [12]. The functions of the products of these gene families are not known, nor is it understood why the genes are highly amplified and localized within roughly distinct subtelomeric neighborhoods.

The *phist* gene family has 65 gene members identified in the genome of the *P*. *falciparum* isolate, 3D7, for which complete sequence information is available (PlasmodDB database, www.plasmodb.org). The encoded PHIST proteins are unified by possessing a single domain, termed PHIST, which is approximately 150 amino acids (aa) long and is predicted to be composed solely of alpha helices [12]. The alpha helical structure was recently confirmed by resolving the crystallographic structure of a *P*. *falciparum* PHIST protein [13]. The aa composition of the domain is extremely divergent, such that paralogous PHIST proteins typically share less than 30% aa identity, with conserved residues largely confined to hallmark tryptophan and other bulky aromatic residues. Grouping of amino acid affinities, as well as analysis of overall gene architectures, reveals three subgroups of PHIST domains, termed PHISTa, b and c [12]. The domain subtypes might indicate functional or parasite stage of expression differences, such as the observed prevalence of an a-type PHIST domain in gametocyte expressed *phist* genes [14], or it might simply reflect the amplification history and thereby sequence relatedness of the gene family. The profound sequence diversity and breadth of the protein family suggests a simple common structure, and possibly function, which tolerates great latitude in sequence. Despite the extreme divergence within the PHIST protein repertoire it is thought that antibody-mediated immune pressure has not shaped diversity; and accordingly, the proteins are localized within the erythrocyte cytoplasm. The related *resa-like* gene family encodes proteins in which a DnaJ domain is fused following a single PHISTb type domain, and is represented by the family flagship protein, RESA [15]. Annotation of the *P*. *falciparum* genome revealed that 6 additional *resa-like* genes are encoded by the parasite. Both the PHIST domain and RESA-like proteins possess a signal peptide sequence, often recessed from the N-terminus, followed by a motif, termed PEXEL or HT, which mediates protein targeting to the erythrocyte cytoplasm [16,17]. PEXEL/HT proteins are proteolytically processed within the motif sequence by an endoplasmic reticulum (ER) resident protease, plasmepsin V [18]. The N-terminally acetylated mature protein is then recognized by transport machinery within the parasitophorous vacuole membrane, facilitating transport to the erythrocyte cytoplasm [19].

The function of the PHIST and RESA-like proteins has not been determined; moreover, it is not known if a single function underpins all proteins, or if the breadth of the repertoire suggests selection for multiple functions. Eleven *phist* and 2 *resa-like* genes were singly disrupted in the course of a project seeking to knockout each of a panel of proteins that are predicted to be exported to the erythrocyte [20]. Of these gene knockout parasite lines, 8 displayed no phenotype in asexual stages, whereas knockout of 5 genes suggested a role for PHIST and RESA-like proteins in trafficking or function of the major parasite virulence protein, the erythrocyte surface protein PfEMP1, or in determination of rigidity of the infected erythrocyte. Analyses of the PfEMP1 intracellular domain (ATS) identified the PHIST domains of PFI1780w and PFE1605w as interactions partners of the ATS domain [13, 21]. The authors hypothesized that the PHIST domain facilitates protein interactions by serving as interaction modules linking parts of the parasite intra-erythrocytic protein network. The erythrocyte cytoskeletal protein Band 4.1 was also shown to interact with a PHIST protein in a yeast two hybrid screen [22]. It would be of interest to extend these analyses to include other PHIST proteins in order to determine if the proposed interactions (reviewed in [23]) are broadly conserved within the PHIST domain family, as well as to identify PHIST interactions in other *Plasmodium* species which do not possess ATS domains or knob structures. In *P*. *falciparum* a PHIST protein (Mal7P1.172) was localized to Maurer’s clefts [20]; whereas study of a PHIST protein in *P*. *vivax* and *P*. *cynomolgi* suggests that the protein resides in caveola-vesicle complexes, termed Schüffner’s dots, which are parasite-derived modifications of the infected erythrocyte [24]. This localization points to possible diversity of PHIST protein function, since *P*. *falciparum* and *P*. *berghei* rodent malaria parasites do not induce Schüffner’s dots-like structures in infected erythrocytes. A recent study showed that a PHISTc protein, named LyMP, localizes to areas in between the membrane knobs at the erythrocyte membrane, and that its C-terminus, but not the PHIST domain, associates directly with the RBC cytoskeleton. Knockout of LyMP reduced adhesion of infected erythrocytes to CD36 by 55%, but did not affect the formation of knobs or the normal distribution of PfEMP1 at the erythrocyte surface [25]. Its mechanism of action remains unknown.

Toward understanding the role of PHIST domain and RESA-like proteins in the *Plasmodium* parasite, we analyzed the expression pattern of *phist* and *resa-like* genes and performed localization studies of epitope-tagged PHIST proteins. In contrast to the great amplification of *P*. *falciparum phist* genes, the rodent malaria parasite *P*. *berghei* possesses 2 predicted functional PHIST domain-encoding genes and 1 pseudogene, and no *resa-like* genes. We thus exploited it as a possible reductionist model system for PHIST domain protein function by performing targeted gene deletions and phenotypic analysis of knockout parasites. Using indirect immunofluorescence assays we observed that both *P*. *falciparum* and *P*. *berghei* PHIST proteins localize within punctate vesicle-like structures in the cytoplasm of infected erythrocytes.

## Materials and Methods

### Parasites and mosquitoes

The *P*. *falciparum* NF54 isolate [26] was cultivated *in vitro* using RPMI 1640 medium supplemented with 10% heat-inactivated human serum as described [27]. Cultures were synchronized by the isolation of schizonts using a Percoll-sorbitol gradient, and placed back into culture [28]. To collect parasites throughout an intraerythrocytic developmental cycle, parasites were harvested at different time points after erythrocyte invasion, pelleted by centrifugation and lysed with 0.1% saponin in PBS. Thin smears of blood on glass slides were made at each time point and stained with Giemsa reagent for microscopy. Gametocytes were prepared by extended *in vitro* culturing of asexual stage parasites, and analyzed when the cultures consisted largely of stage IV and V parasites. The isolates Dd2, HB3 and IT4 [29–31] were cultivated as described above.

The gametocyte-producing reference clone, cl15cy1 (HP), of the ANKA strain of *P*. *berghei* was maintained and gametocytes produced as described [32] and the non-gametocyte-producing clone (HPE) of the ANKA strain was also used in some experiments [33]. To collect parasites from different stages of the intraerythrocytic developmental cycle, synchronized blood stage infections of *P*. *berghei* HPE clone were performed *in vivo*, in Swiss Webster mice, as described [34]. Thin smears of blood on glass slides were made at each time point and stained with Giemsa reagent for microscopy. *Anopheles stephensi* mosquitoes were reared as described [34]. *P*. *berghei*-infected mosquitoes were maintained at 19°C. Mosquito stages were obtained after feeding 3 to 5 day-old *An*. *stephensi* mosquitoes on Swiss Webster mice that were infected with either *P*. *berghei* ANKA HP or *phist* gene knockout clonal lines. Oocyst samples for RT-PCR consisted of infected midguts that were removed 5, 10 and 15 days post infection. To determine parasite development in the mosquito, oocysts were counted from midguts removed at day 10 post infection; midgut sporozoites were counted on day 14 post infection; and salivary gland sporozoites were quantified from dissected salivary glands on day 17–21 post infection. Live sporozoites for *in vitro* and *in vivo* assays were isolated from day 21 salivary glands as described [32].

### Animals

All experiments using animals were approved by the Weill Cornell Medical College Institutional Animal Care and Use Committee (WCMC IACUC) under the Animal Protocol number 0704-613-A, or by the New York University School of Medicine Institutional Animal Care and Use Committee (NYU IACUC) under the Animal Protocol number 080413–02, and were performed in accordance with the applicable guidelines and regulations. Animals were housed in the Weill Cornell Medical College animal facility or in the New York University School of Medicine animal facility, where they received daily care and husbandry.

Six week old female Swiss Webster mice, female C57Bl/6 mice and male Wistar Kyoto rats were obtained from Taconic Biosciences, Inc. For *P*. *berghei* infection, mice and rats were injected intraperitoneally or by mosquito bite inoculations as described [32]. To generate *phist* gene knockout parasites, *P*. *berghei* ANKA HP for transfection was collected from Wistar rats that harbored infections between 0.5 to 3% parasitemia. Parasitemia was monitored daily by tail vein blood smears. For collection of infected blood, animals were terminally exsanguinated by cardiac puncture, followed by cervical dislocation. Animals were euthanized once experimental data was collected or if clinical symptoms of cerebral malaria were observed. Twelve week old New Zealand White rabbits (Charles River Laboratories International, Inc.) were used for production of immune sera.

### RNA isolation and transcript expression analysis by real-time quantitative RT-PCR

RNA was isolated using the Trizol-based method (Invitrogen) according to the manufacturer’s instructions, treated with DNase I (Invitrogen), and purified on RNeasy MinElute Cleanup columns (Qiagen). RNA was reverse-transcribed using Superscript II that was primed with random hexanucleotides (Invitrogen). Real-time quantitative RT-PCR was performed using an ABI Prism 7900HT sequence detector (Applied Biosystems). Reactions were prepared in 20 μL volumes using a SYBR Green PCR master mix (Applied Biosystems) and 1 μM gene-specific primers. The absence of genomic DNA contamination was confirmed by PCR amplification on sham-treated RNA samples that lacked reverse transcriptase. The specificity of amplification for each PCR product was confirmed by dissociation curve analysis. The efficiency of amplification was verified by using different concentrations of genomic DNA as templates in order to calculate the median cycle threshold (CT) value for each primer pair. All primer pairs included in this study displayed the same median CT value. Relative quantification of cDNA was performed using the standard curve method (User Bulletin 2, ABI, http://www.appliedbiosystems.com) to determine the efficiency of the target and reference amplification and to quantify cDNA in each sample. The normalized expression for each gene was determined as the ratio: relative amount of target gene cDNA/ relative amount of control gene cDNA. Triplicate PCR reactions were analyzed for each sample. Transcript expression of *P*. *falciparum* genes was normalized to the expression of the control gene, *arginyltRNA synthetase* (*PFL0900c*), while the transcript expression of *P*. *berghei* genes was normalized to the expression of the control gene, *Pbhsp70* (*PBANKA_091440*). Gene-specific primers were empirically designed for select *P*. *falciparum* PHIST domain-containing genes and the *P*. *berghei* genes, *PBANKA_114540* and *PBANKA_122900* (see S1 Table for primer sequences).

### Epitope-tagged constructs to fuse 3 c-myc epitopes at the C-terminus of *phist* genes

The sequences of all PCR primers used to make constructs in this study are included in S2 Table. Epitope-tagged constructs for episomal expression were designed to fuse DNA encoding 3 c-myc epitopes at the C-terminus of the *P*. *falciparum* genes, *PFE1600w* and *PFE1605w*. The coding sequences corresponding to both genes were amplified by PCR from genomic DNA template of the *P*. *falciparum* NF54 isolate using the following primer pairs: PFE1600wSXhoI plus PFE1600wASBglII; and PFE1605wSXhoI plus PFE1605wASBglII. The PCR products were digested with *Xho*I and *Bgl*II and cloned into the expression vector, pHL-dhfr-3myc, which is a derivative of the pHLH1 vector [35]. pHL-dhfr-3myc contains 3 consecutive c-myc epitopes within an expression cassette that is driven by the *P*. *falciparum* 5′ *hrp3* promoter and the 3′ *hrp2* terminator to ensure a high expression level of the *phist* genes; and an hDHFR expression cassette for selection of transformed parasites with pyrimethamine. Expression vectors were introduced into the *P*. *falciparum* isolate NF54 via the erythrocyte-loading method [36]. Stable transformant parasites were selected by culturing in media containing 40 ng/ml pyrimethamine. Episomal gene expression was detected by reverse transcription and real-time quantitative PCR using specific primers PFE1600wS2 plus PFE1600wAS2; PFE1600wS2 plus PFE1600wmycAS3c; PFE1605wS2 plus PFE1605wAS2 and PFE1605whrpS3c plus PFE1605wAS2, following the protocol described in the above transcript expression analysis section. As a control for expression and localization experiments we used the pHL-dhfr-luciferase line (a gift from Kirk Deitsch), which expresses the luciferase protein and is a derivative of the pHLH1 vector [35]. To ensure that parasites were expressing the correct protein, plasmid was rescued by transforming chemically competent *Escherichia coli* with 100 ng of purified genomic DNA from selected transformant parasites, and plasmid nucleotide sequence was determined.

Two independent lines of transgenic parasites were established for each construct. In the transgenic populations the average copy number per parasite of the introduced chimeric *PFE1600w* and *PFE1605w* genes was determined, by quantitative real time PCR, to be approximately 3 to 4 copies (S3B Fig). Transformed parasite lines which had been in culture for more than 100 generations prior to transgene quantification (termed Line 1 in S3B Fig), exhibited variation in the average number of gene copies between generations, while parasite populations that had been kept in culture for no longer than 30 generations (termed Line 2 in S3B Fig) displayed homogeneous copy number from one generation to the next (S3B Fig). This might be due to imperfect plasmid duplication and segregation during schizogony, such that the population becomes increasingly heterogeneous as it is propagated for many generations. Transcript levels of endogenous and episomal genes reached the maximum level of expression at similar intraerythrocytic stages as determined by quantitative real time RT-PCR, suggesting that the expression vector *hrp3* promoter activity, at the late ring stage, drove transgene expression coincident with the peak of expression of endogenous *PFE1600w* and *PFE1605w* (S3C Fig). However, expression of episomal *PFE1600w* and *PFE1605w* are 10- and 22-fold higher, respectively, than the cognate endogenous genes. Western blot analysis of the epitope-tagged proteins using anti-c-myc antibodies showed expression of proteins having the expected molecular weights, with no additional bands that might suggest cross-reactivity or protein processing (S3D Fig). Phenotypic analysis of parasites harboring the *PFE1600w-c-myc* or *PFE1605w-c-myc* expression vectors exhibited normal growth in comparison to wild type (wt) NF54, indicating that over-expression of these PHIST proteins are not toxic to the parasite (data not shown).

### Generation of transgenic *P*. *berghei* ANKA parasites expressing mCherry-tagged PBANKA_136550

Transfection of *P*. *berghei* parasites, selection and cloning of the transgenic parasite line expressing mCherry-tagged PBANKA_136550 (IBIS1) was performed as described [37]. To generate the targeting vector, the *smac* targeting region within plasmid pL1419 [38] was replaced by the targeting region of PBANKA_136550. This region was amplified with the primers 6288 and 6289 (S2 Table). Transfection of parasites with this construct results in tagging of the endogenous gene with mCherry by a single cross-over integration of the construct. Correct integration at the chromosome 13 locus was determined by Southern blot analysis of digested genomic DNA or chromosomes separated by pulse-field gel (PFG) electrophoresis and hybridization with a probe corresponding to the *P*. *berghei* 3’UTR of *dhfr/ts*.

### Antibodies

For immunofluorescence microscopy the following antibodies were used: anti-c-myc monoclonal antibody FITC conjugate (Sigma-Aldrich F2047); rabbit anti-SBP1 rabbit polyclonal antibody directed against the N-terminus of SBP1 (a gift from Dr. Brian Cooke; [39]); anti-TER-119-FITC labeled antibody (eBioscience); goat anti-mouse Alexa 488-conjugate IgG (Molecular Probes); goat anti-rabbit Alexa 488-conjugate IgG (Molecular Probes); and goat anti-rabbit Alexa 594-conjugate IgG (Molecular Probes).

For the generation of anti-*P*. *berghei* PHIST polyclonal antibodies, 30-mer peptides corresponding to the repeat region of PBANKA_114540 (CSEKKSEKKSEKKNEENSEKKSKKKSEKKN) and PBANKA_122900 (CPLKPEQEENVDPLKPEQEENIKPLKPEQK) were synthesized by the Proteomics Resource Center of the Rockefeller University with an Applied Biosystems Model 430A instrument using FMOC based chemistry. Peptides were conjugated to mariculture keyhole limpet hemocyanin (mcKLH) using the kit Imject Maleimide Activated mcKLH (Pierce 77611) according to the manufacturer’s instructions. Twelve weeks old New Zealand White rabbits (Charles River) were immunized with 100 μg of conjugated peptide in Freund’s Complete Adjuvant (Sigma-Aldrich), followed by 3 boosts with 50 μg of conjugated peptide in Freund’s Incomplete Adjuvant (Sigma-Aldrich). One rabbit was immunized with each peptide. Peptides were covalently immobilized in a beaded agarose support using the SulfoLink kit (Pierce 44895) and the resulting columns were used to affinity purify antibodies specific to PBANKA_114540 and PBANKA_122900.

For Western blot analyses, anti-c-myc monoclonal antibody (Sigma-Aldrich M4439); anti-mouse IgG peroxidase conjugated (Sigma-Aldrich A9044) and anti-rabbit IgG peroxidase conjugate (Sigma-Aldrich A0545) secondary antibodies were used.

### Indirect immunofluorescence assay and immunoelectron microscopy

Parasite culture or mouse tail vein blood was spotted within the wells of teflon-coated microscope slides (Electron Microscopy Sciences, PA), air dried and fixed for 10 min in cold methanol. For blocking of nonspecific binding, the fixed cells were incubated in PBS containing 2% bovine serum albumin and 10% normal goat serum for 1 h at room temperature. The preparations were then incubated for 1 h at room temperature with the primary antibody at the following concentrations: 5 μg/ml anti-c-myc monoclonal antibody FITC conjugate; 1:500 rabbit anti-SBP1 polyclonal antibody; 2 μg/ml affinity purified rabbit anti-PBANKA_114540; and 2 μg/ml affinity purified rabbit anti-PBANKA_122900. Slides were washed in PBS and incubated for 1 h at room temperature with appropriate secondary antibody at the following concentrations: 1 μg/ml goat anti-mouse Alexa 488-conjugate IgG; 1 μg/ml goat anti-rabbit Alexa 488-conjugate IgG; 1 μg/ml anti-rabbit Alexa 594-conjugated IgG. Nuclei were stained with DAPI. Samples were analyzed in an Olympus BX-51 fluorescence microscope with an Optronics digital imaging system.

For analysis of mCherry expression of the transgenic lines, live parasites were examined by microscopy using a Leica DMR fluorescent microscope with standard FITC and Texas Red filters. Parasites nuclei were labeled by staining with 2 μmol/L Hoechst-33258 and erythrocyte surface membranes were stained with the 1:200 anti-mouse TER-119-FITC labeled antibody at room temperature for 30 min and washed with 500 μL of RPMI-1640 medium.

For confocal microscopy, *P*. *falciparum* infected erythrocytes were washed twice in PBS, fixed in 4% paraformaldehyde, 0.075% glutaraldehyde (Electron Microscopy Sciences, PA) in PBS for 20 min at room temperature, washed 3 times in PBS and spread on poly-L lysine-coated glass slides. Slides were washed twice in PBS and incubated in 0.15% glycine in PBS for 10 min at room temperature. After 3 washes in PBS, cells were permeabilized for 10 min in 0.1% Triton X-100 in PBS, and washed twice in PBS. Samples were blocked and incubated with antibodies as described above, and analyzed in a Zeiss Exciter LSM500 confocal microscope system. All indirect immunofluorescence experiments included secondary antibodies alone controls.

### Western blot analysis

Infected erythrocytes were lysed in SDS sample buffer and proteins separated by SDS-PAGE and transferred to Hybond P membrane (Amersham Biosciences) according to the manufacturer’s instructions. The membranes were blocked for nonspecific binding by incubation in Tris-buffered saline (TBS) containing 5% skim milk followed by incubation for 1 h at room temperature with 2 μg/ml anti-c-myc monoclonal antibody, or 1:1,000 anti-Pf39 polyclonal antibodies. After washing in TBS containing 0.1% Triton X-100, the membranes were incubated for 1 h at room temperature with 1:80,000 anti-mouse IgG peroxidase conjugated (Sigma-Aldrich A9044).

### Targeted disruption of *P*. *berghei* phist genes

The sequences of all PCR primers used in this study are included in S2 Table. *PBANKA_122900* knockout (KO) parasites were generated by double homologous recombination using the targeting vector b3D.DT^H.^D (available from MR4; http://www.malaria.mr4.org) and *P*. *berghei* ANKA erythrocytic stages. Two fragments of 732 and 626 bp (shaded in gray in S5A Fig) were amplified by PCR from *P*. *berghei* ANKA genomic DNA using the following specific primer pairs: PB848ko5SKpnI plus PB848ko5ASHindIII; and PB848ko3SEcoRI plus PB848ko3ASBamHI. To derive the disruption plasmid these PCR fragments were sequentially cloned into the b3D.DT^H.^D vector using the *Kpn*I/*Hind*III sites, followed by insertion into the *Eco*RI/*Bam*HI sites. The disruption vector was then digested with *Kpn*I and *Bam*HI to release the fragment for transfection. *P*. *berghei* ANKA schizonts were collected from 2 Wistar rats and electroporated with 10 μg of DNA using the Amaxa Nucleofector (program U33) as described [37]. Selection and cloning by limiting dilution were performed in mice as described [40]. Integration of the transfected DNA at the correct location was verified by both diagnostic PCR and Southern blot analysis. PCR was performed with 100 ng of genomic DNA from wt or *PBANKA_122900* KO parasites for 35 cycles and annealing temperature of 50°C using the primers a; b (identical to PB848ko5ASHindIII); b’; c (identical to PB848ko3SEcoRI); c’; and d (locations of primers shown in S5A Fig). Southern blot analysis was performed with 10 μg of genomic DNA from wt or *PBANKA_122900* KO parasites, digested with *Cla*I and *Eco*RI. A 732 bp hybridization probe was amplified with the primers PB848ko5SKpnI and PB848ko5ASHindIII. The probe was labeled with digoxigenin and hybridization was performed at 48°C using DIG High Prime DNA Labeling and Detection Starter Kit II (Roche Diagnostics), according to the manufacturer’s instructions. Lack of *PBANKA_122900* transcripts in the *PBANKA_122900* KO clones was confirmed by RT-PCR, using RNA that was isolated from schizonts. The synthesis of cDNA is described in the transcript expression analysis section, and PCR was performed for 35 cycles of amplification using the oligonucleotides “e” plus “f” and “g” plus “h”.

Two fragments of 744 bp and 638 bp length corresponding to the gene *PBANKA_114540* (shaded in gray in S4 Fig) were amplified by PCR using as template genomic DNA from *P*. *berghei* ANKA parasites and the primer pairs: PB106ko5SHindIII plus PB106ko5ASPstI; and PB106ko3SKpnI plus PB106ko3ASEcoRI. These PCR fragments were sequentially cloned into the restriction sites *Hind*III/*Pst*I and *Kpn*I/*Eco*RI of the plasmid pDEF-hDHFR. This gene targeting disruption plasmid was digested with *Hind*III and *Eco*RI to release the fragment for transfection. Transfection of *P*. *berghei* schizonts was performed as described for the *PBANKA_122900* gene KO. Using this construct and gene disruption procedure we were unable to knockout *PBANKA_114540* in 3 independent experiments.

### Prepatent period and asexual multiplication of erythrocytic stages *in vivo*

To determine the prepatent period, 10^2^ to 10^4^ salivary gland sporozoites from *P*. *berghei* ANKA wt or *PBANKA_122900* KO lines were resuspended in RPMI and injected intravenously in the tail vein of naive 6 weeks old female Swiss Webster mice. At 24 h intervals after sporozoite injection, blood samples were withdrawn from the tail of injected animals and analyzed on Giemsa reagent-stained thin smears for the presence of parasites. The prepatent period was assessed by determining the number of days between sporozoite injection, and the time when at least 3 to 5 parasites could be detected by analyzing a minimum of 10,000 erythrocytes on Giemsa reagent-stained blood smears on glass slides. Two independent experiments were performed, using 5 mice per experiment. After prepatent period data collection, mice were euthanized.

To analyze the asexual multiplication of erythrocytic stages, 6 weeks old female Swiss Webster mice were injected intravenously with 2 x 10^5^ mature schizonts of either *PBANKA_122900* KO or *P*. *berghei* ANKA wt parasites. Survival of the mice was monitored and parasitemia was determined by Giemsa reagent-stained thin blood smears collected at 24 h intervals. A similar experiment was performed by injecting 10^3^ and 10^4^ asexual stage parasites intravenously into 6 weeks old female C57Bl/6 mice. All experiments were performed twice with 5 mice per group per experiment. At the onset of clinical malaria symptoms mice were euthanized.

### Sporozoite invasion and development assays

Hepa 1–6 cells were used for quantification of sporozoite invasion as described [32], using *PBANKA_122900* KO or *P*. *berghei* ANKA wt sporozoites based upon double staining with a monoclonal antibody (mAb) 3D11, directed against the repeat region of *P*. *berghei* CSP in an assay that distinguishes intracellular from extracellular sporozoites [41, 42]. To quantify exoerythrocytic forms development *PBANKA_122900* KO or *P*. *berghei* ANKA wt sporozoites were assayed for development in Hep 1–6 cells as described [32]. Experiments were performed in triplicate.

## Results

### The P. falciparum phist gene family

The *phist* gene family was identified in the course of describing the repertoire of *P*. *falciparum* genes that encode proteins harboring PEXEL/HT motifs predicting export to the erythrocyte cytoplasm [12]. We revisited description of the published *phist* gene catalog and gene structures (see Fig 5 of Sargeant *et al*. [12]), in order to update the observation that 19 of the genes encoding PHIST domain proteins lacked predicted signal peptides and PEXEL/HT motifs, perhaps due to insufficient sequence information for gene models at the time of that publication. The current version of PlasmoDB (September 2015) matches our in-house annotation for all identified *phist* genes within the genome of the *P*. *falciparum* 3D7 isolate accessible at PlasmoDB and GenBank; which was arrived at via exhaustive iterative BLAST analyses, as well as annotation of all open reading frames within sub-telomeric regions and internal clusters of PEXEL/HT encoding genes. Indeed, the gene models for all 19 of the above genes were corrected, to show the presence of a signal peptide plus PEXEL/HT motif in 14 instances, and confirmation of the remaining genes as likely pseudogenes (compendia and details of methods in S1 Fig; detailed in S3 Table). Two of the 65 genes are possible pseudogenes (namely, *MAL13P1*.*11* and *PF14_0764*), and additional predicted *phist* pseudogenes are listed in the inset of S1 Fig. The 7 *resa-like* genes were all confirmed [12] and no gene structure revisions were required, and no additional *resa-like* genes were identified. For both *phist* and *resa-like* genes 2-exon gene structures predominate, in which the signal peptide is encoded on the first exon and the PEXEL/HT motif is adjacent within the second exon. However, 6 *phist* genes are encoded by 3-exon gene structures, and 3- and 4-exon gene structures are also common in the predicted pseudogenes. A few of the *phist* genes are almost identical and might be indicative of subtelomeric blocks of duplicated DNA [2, 43]; for example, *PFF0075c* and *PFF1510w* on the left and right arms, respectively, of chromosome 6. In this study we did not revisit the nomenclature dividing the *phist* gene family into subfamilies, termed a, b and c [12], and such designations in S1 Fig.

A comparison of all PHIST proteins within the *P*. *falciparum* isolate nucleotide sequence databases (HB3, Dd2, RO-33/Ghana, D6, D10, VS/1, K1/Thailand, Santa Lucia, FCC-2/Hainan and Senegal V34.04; Broad Institute; downloaded for local TBLASTN analyses from the NCBI Trace Archive; as well as SNP data from the MalariaGen Pf3k project, www.malariagen.net/apps/Pf3k) indicates that the orthologous members from each isolate are 99–100% conserved (data not shown; [44]), despite the extensive sequence divergence between paralogs within the PHIST domain family, which typically possess less than 30% identity. This indicates that PHIST proteins likely diversified in response to a selective force other than immune pressure, as opposed to what is observed for the *var* genes, where orthologs are found to be highly divergent between field or laboratory isolates. This observation is in agreement with their predicted functional localization within the erythrocyte cytoplasm.

### Transcript level analysis of *P*. *falciparum phist* and *resa-like* genes

**Fig 1:**
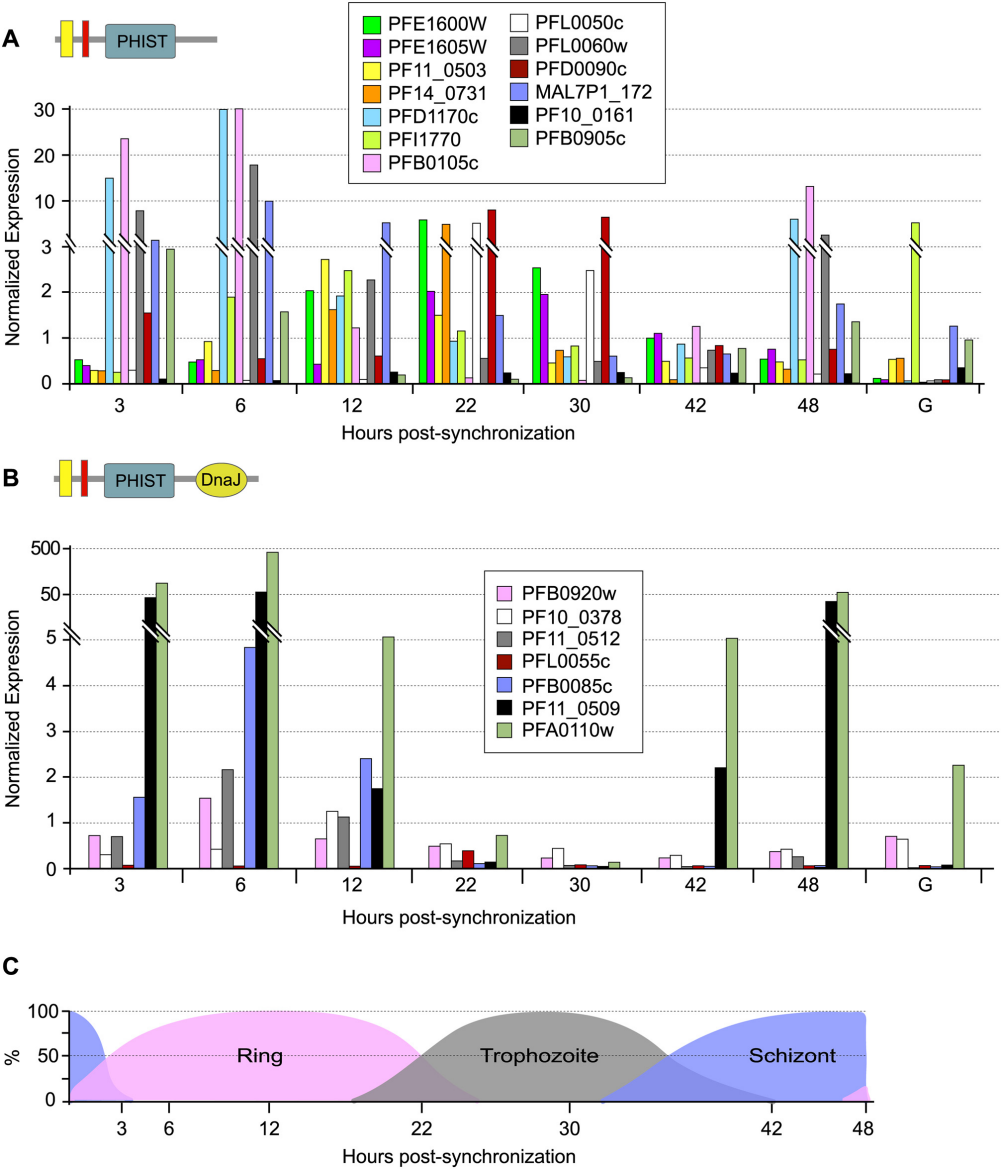
Transcript expression analysis of *phist* and *resa-like* genes during *P*. *falciparum* intraerythrocytic stages. Gene-specific transcript levels for select *phist* genes **A**) and for the *resa-like* genes **B**) were analyzed by real time PCR using cDNA prepared from intraerythrocytic synchronized asexual and gametocyte *P*. *falciparum* cultures. Note that *resa* (*PFA0110w*) and *resa2* (*PF11_0509*) are highly expressed relative to the other *resa-like* genes. Transcript expression was normalized to the expression of the control gene *arginyl tRNA synthetase* (*PFL0900c*). Hours post-synchronization indicate time in hours after adding purified schizonts to fresh blood culture. In the schematic depiction of *phist* and *resa-like* genes, yellow represents the signal sequence, and red represents the PEXEL/HT motif. The life cycle stage percentage in the population for each time point is shown in **C**). The letter “G” indicates mature gametocytes.

**Fig 2:**
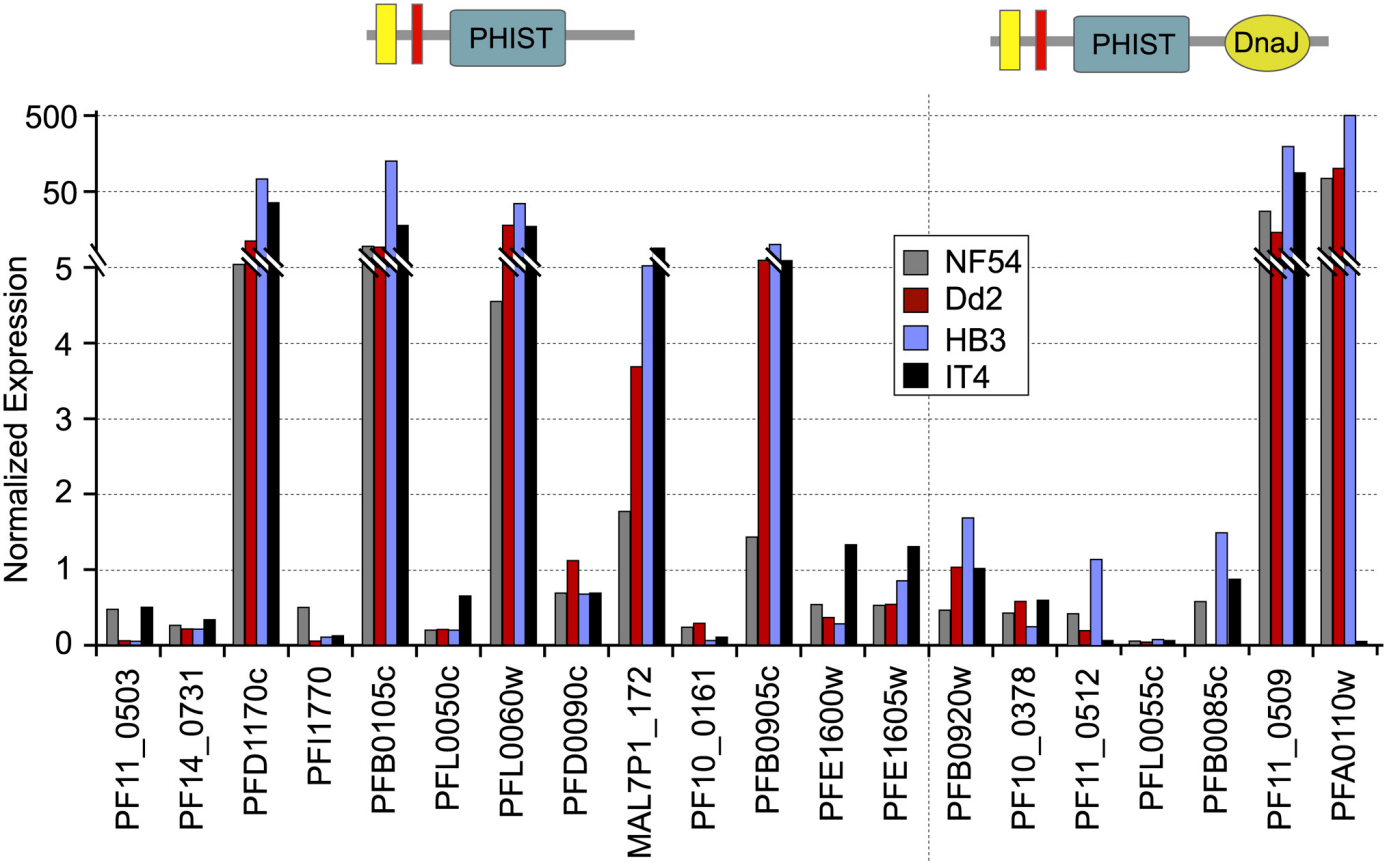
Transcript expression analysis of *phist* and *resa-like* genes in 4 *P*. *falciparum* isolates. The transcript levels of select *phist* and *resa-like* genes in 4 isolates of *P*. *falciparum*, NF54, Dd2, HB3 and IT4, were compared by real time PCR using cDNA prepared from late schizont stage parasites. Transcript expression was normalized to the expression of the control gene *arginyl tRNA synthetase* (*PFL0900c*). In the schematic depiction of *phist* and *resa-like* genes, yellow represents the signal sequence, and red represents the PEXEL/HT motif.

To determine the transcript expression profiles of the *phist* and *resa-like* gene repertoire during the *P*. *falciparum* life cycle, we exploited the high-throughput gene expression data obtained for the asexual intraerythrocytic developmental cycle of *P*. *falciparum* accessible at PlasmoDB. Thirteen *phist* genes were selected as candidates for further studies, with the criteria of high expression levels but differing in the timing of expression, plus the 7 *resa-like* genes (available expression data shown in S2 Fig). Real time RT-PCR assays of these select genes yielded transcript expression profiles throughout the asexual intraerythrocytic developmental cycle (Fig 1) similar to the high-throughput transcriptome data, thus validating the expression patterns of the *phist* and *resa-like* genes described in PlasmoDB. For the 13 *phist* genes expression levels span a wide range and different stages of the asexual cycle, including transcription of a subset detectable in gametocytes [14]. This expression pattern might support the hypothesis of functional diversification of PHIST proteins, underpinned by multiple clusters of gene expression, where induction of subsets of PHIST genes occur at specific developmental stages (e.g. rings, trophozoites, and schizonts) when their products are required (examples are indicated by the red and blue highlighted genes in S2 Fig). We also compared the transcript levels of the select *phist* genes and the 7 *resa-like* genes in 4 isolates of *P*. *falciparum*, NF54, Dd2, HB3 and IT4 (Fig 2). Since the life cycle length differs slightly among these isolates, we chose to analyze late schizont stage parasites matched for similar developmental morphology in each isolate. All genes analyzed were expressed in the 4 isolates and their transcription levels at the schizont stage were sufficiently similar to indicate that expression of the PHIST and RESA-like protein repertoire is consistent in intraerythrocytic development across *P*. *falciparum* isolates.

### Protein structure, transcript level and cellular localization of two *P*. *falciparum* PHIST proteins

**Fig 3:**
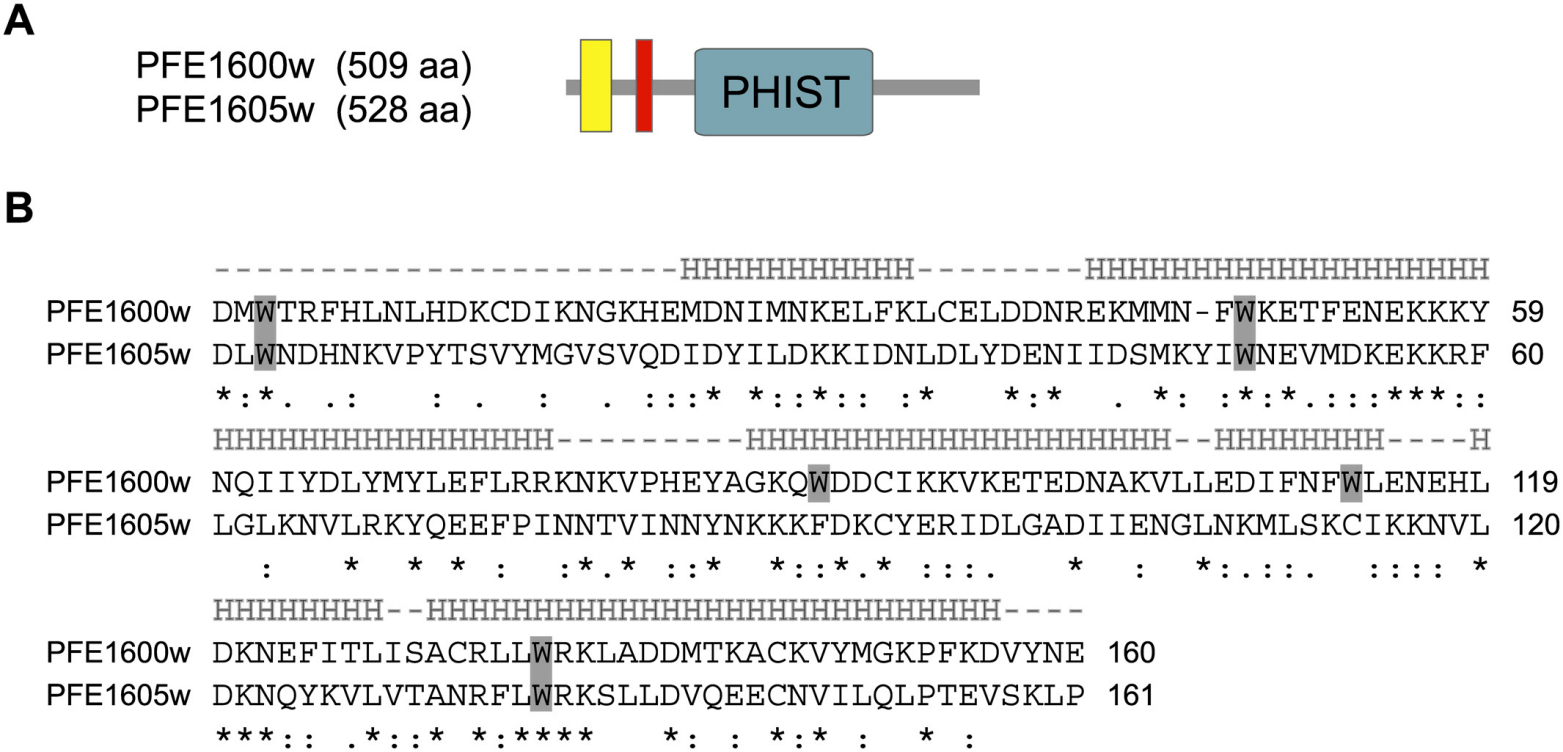
Protein structure of the *phist* genes *PFE1600w* and *PFE1605w*. **A**) PFE1600w and PFE1605w possess a similar protein architecture, composed of a recessed signal peptide sequence (yellow rectangle) followed by a PEXEL/HT motif (red rectangle) and a PHIST domain (green rectangle). The numbers in the schematics indicate the lengths in aa for each protein. **B**) Amino acid sequence alignment of the PHIST domains from PFE1600w and PFE1605w. Tryptophan residues are shaded in gray. The predicted helical segments are shown above the alignment, marked by “H”. Below the alignment, stars indicate identical aa residues, 2 dots indicate conserved substitutions and one dot indicates semi-conserved substitutions.

Two *P*. *falciparum phist* genes, *PFE1600w* and *PFE1605w* (the latter recently termed LyMP, [25]), indicated by orange highlight in S1 Fig, were selected for detailed characterization. The genes are located adjacent to each other within the subtelomeric region of the right arm of chromosome 5, and similarly to most *phist* genes, possess a typical 2-exon gene structure (Fig 3A). The PHIST domains of PFE1600w and PFE1605w each span approximately 150 aa and comprises 4 predicted interspersed alpha helices containing conserved tryptophan residues, which are hallmarks of all PHIST domains (Fig 3B). The unifying feature of PHIST domains is likely conserved structure, perhaps including key conserved residues, rather than primary amino acid sequence, as shown in Fig 3B. The 2 proteins share only 21% aa identity (Fig 3B), and it is this dissimilarity that motivated us to choose them for study.

*PFE1600w* is predominantly transcribed in late ring and early trophozoite stages, while transcript levels of *PFE1605w* peak similarly but slightly later (Fig 1A). Low transcript levels of both genes can be detected soon after merozoite invasion and in late trophozoite and schizont stages, while transcripts were not observed in gametocytes.

**Fig 4:**
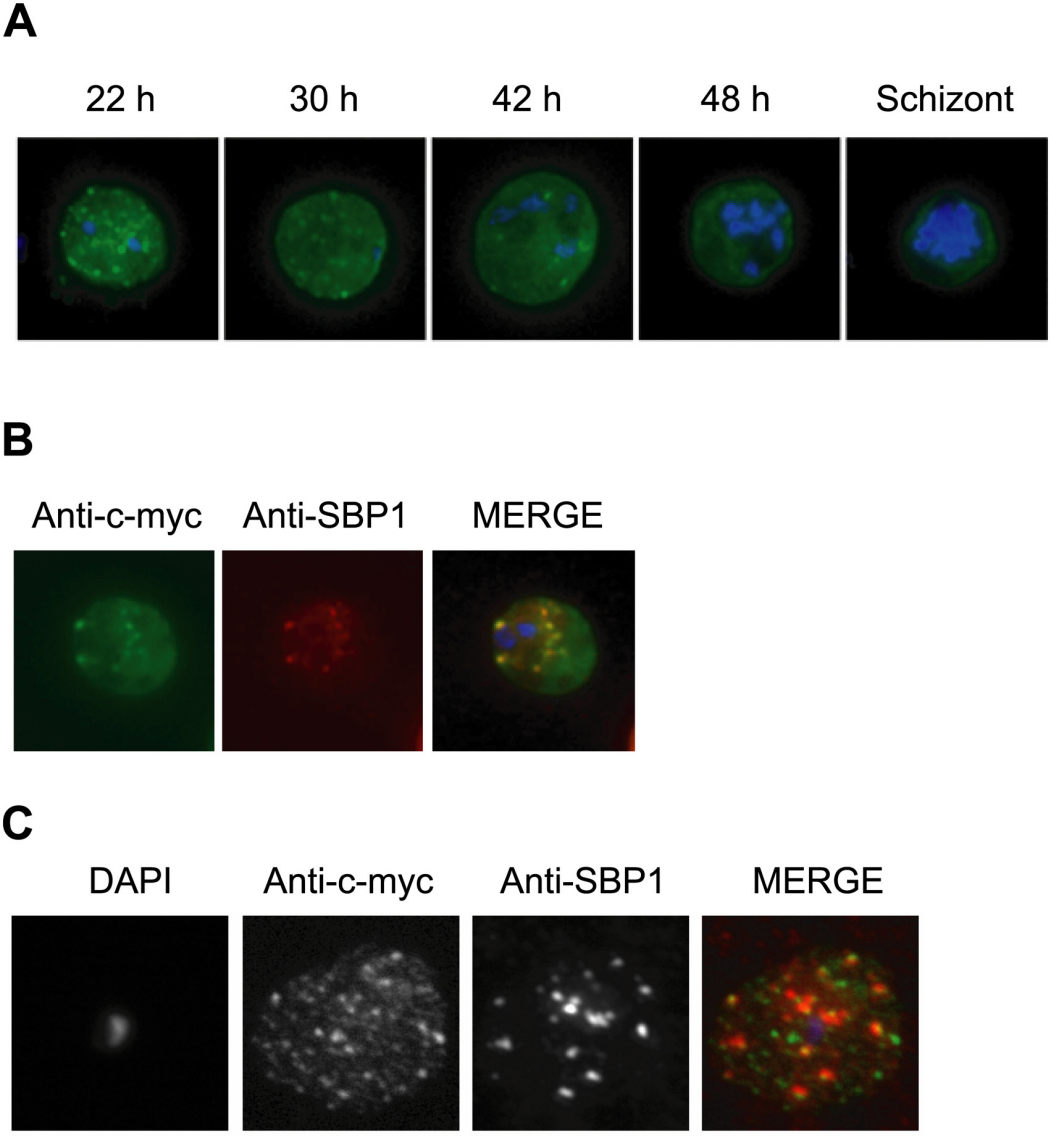
Immunolocalization of c-myc epitope-tagged PFE1605w PHIST protein within infected erythrocytes. **A**) Synchronized parasites were harvested at different time points after Percoll-sorbitol purification, air-dried and fixed with ice-cold methanol. Epitope-tagged proteins were stained with anti-c-myc monoclonal antibodies that were conjugated with FITC. Nuclei were stained with DAPI (blue). The abbreviations used to describe parasite stages are as follows: 22 h, late ring stage; 30 h, early-trophozoite stage; 42 h, mid-trophozoite stage; 48 h, late trophozoite stage; and schizont stage. **B)** Co-localization was assayed using rabbit polyclonal SBP1 followed by goat anti-rabbit IgG conjugated with Alexa 594 (red). **C)** The pattern of expression of c-myc epitope-tagged PFE1605w PHIST protein is dependent on the fixation protocol. When infected erythrocytes are fixed in solution with 1% paraformaldehyde, 0.075% glutaraldehyde, then PHIST protein shows a broader punctate expression than the Maurer’s cleft marker, SPB1.

As a means to understand PHIST protein function, we sought to determine the cellular localization and predicted export of PFE1600w and PFE1605w to the erythrocyte cytoplasm. Epitope-tagged constructs were designed to fuse 3 c-myc epitopes at the C-terminus of PFE1600w and PFE1605w (S3A Fig), and the resulting expression vectors were introduced into the *P*. *falciparum* NF54 isolate and selected for stable episomal expression via pyrimethamine drug pressure, as elaborated in the Materials and Methods section. Epitope-tagged PFE1600w and PFE1605w proteins were localized in synchronized asexual stages by indirect immunofluorescence assay (IFA) using anti-c-myc monoclonal antibodies. Epitope-tagged PHIST proteins were first observed in the ER of mid-ring stage of both PFE1600w and PFE1605w transformed parasite lines, in co-localization with the abundant ER resident protein, Pf39 (data not shown). At the late ring stage, PHIST proteins were observed exported to the erythrocyte cytoplasm, at least in partial co-localization with the marker of Maurer’s clefts, SBP1 (Fig 4A and 4B). PFE1600w was abundantly observed in the ER of late stage parasites (data not shown); and it is possible that this localization represents aberrant trafficking due to over-expression of the transcript and thereby protein, perhaps due to the use of a heterologous promoter. The localization to the erythrocyte cytoplasm validates trafficking that is predicted by the presence of a PEXEL/HT motif. Using a different fixation method, we also observed a broader distribution of a punctate pattern for PFE1605w-myc, having only partial overlap with the SBP1 marker (Fig 4C). PFE1605w was recently shown to be cotranslocated with PfEMP1 within the erythrocyte cytosol [13], in agreement with our localization data for PHIST proteins. Further study, of multiple PHIST proteins, is required to determine the association of PHIST protein with Maurer’s clefts. IFA on non-permeabilized infected erythrocytes failed to detect either of the epitope-tagged PHIST proteins on the erythrocyte surface (data not shown), suggesting a final cellular destination and function within the erythrocyte cytoplasm.

### Structure and transcript levels of *P*. *berghei phist* genes

*P*. *falciparum* and other *Plasmodium* species, such as *P*. *reichenowi* [45] *P*. *vivax* [12] and *P*. *knowlesi* have highly amplified sets of *phist* genes, but rodent malaria parasites have a minimal repertoire. In the *P*. *berghei* genome sequence we identified 3 *phist* genes (namely, *PBANKA_114540*, *PBANKA_122900* and *PBANKA_070080*), via BLAST analysis using PHIST domains and pssm profiles as queries. Our result differs from the single *P*. *berghei phist* gene described in the original annotation [12]. Orthologs of PBANKA_114540 and PBANKA_122900 were identified in the genome nucleotide sequence databases for other rodent malaria parasites; namely, *P*. *yoelii* (PY00289 and PY01786, respectively) and *P*. *chabaudi* (PCAS_114490 and PCAS_122970, respectively). Additional *phist* genes were not identified in rodent malaria parasites. BLAST analyses using the PHIST domain as queries, identify as reciprocal best hits PBANKA_122900, the *P*. *vivax* PHIST protein PvPHIST/CVC-81_95_ (PVX_093680), and *P*. *falciparum* PF08_0137. This indicates possible orthologous (vertically inherited) relationships, as recently proposed by Akinyi *et al*. [24]; and is supported by the observed synteny of adjacent genes, including the ookinete-expressed gene *warp*, together composing a locus which is conserved across the *Plasmodium* genus. *PBANKA_122900* is internally localized in *P*. *berghei*, but the locus appears to be sub-telomeric in species other than rodent malaria parasites. Orthologous conservation across the *Plasmodium* genus might imply functional conservation underpinned by the PHIST domain, as discussed further in the Discussion section.

**Fig 5:**
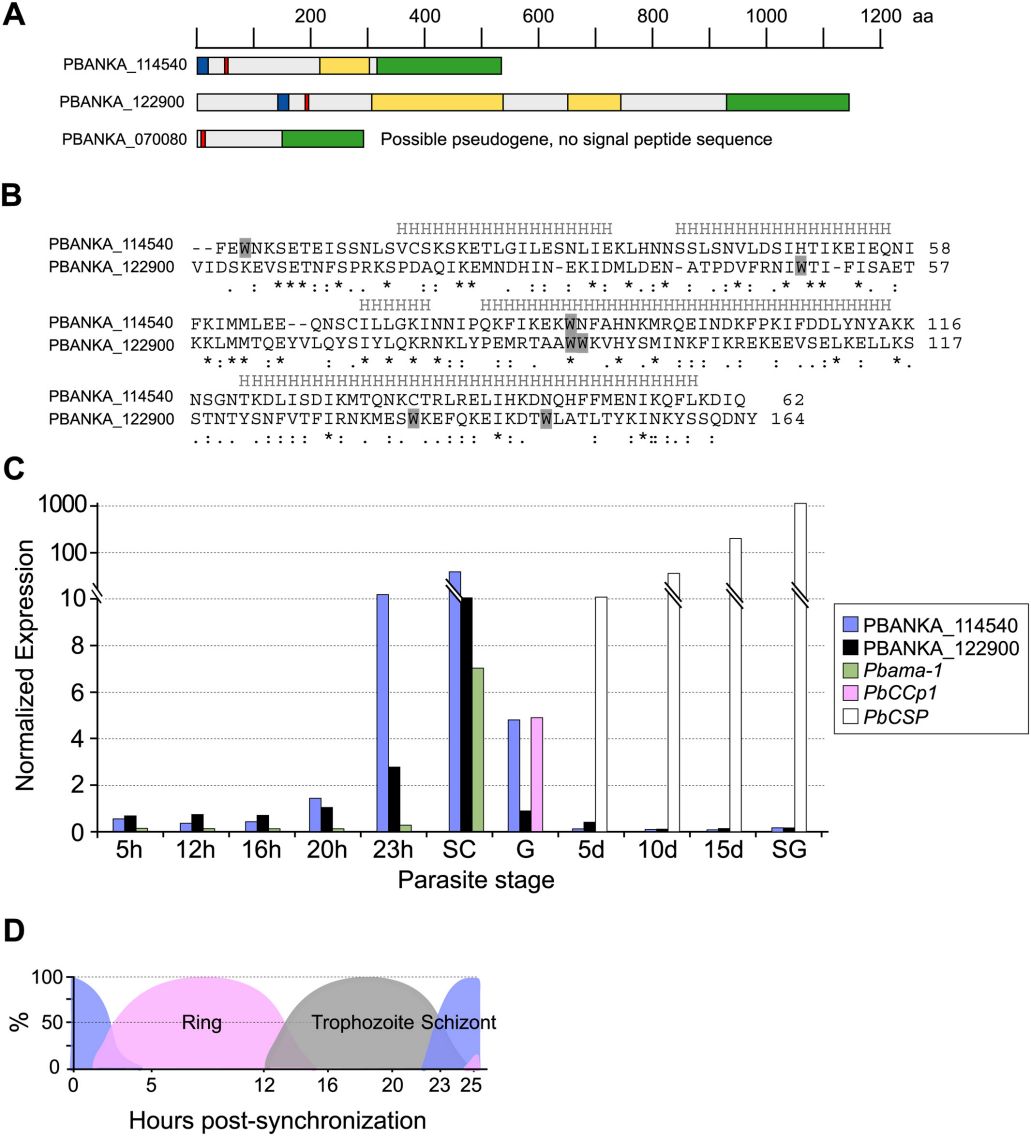
The *P*. *berghei phist* genes. **A**) Protein domain architectures for *P*. *berghei* PHIST-domain encoding genes. The ruler above the architectures indicates protein lengths in aa, drawn to scale. Blue boxes represent the signal sequence; red boxes represent the PEXEL/HT motif; green boxes indicate the PHIST domain; and yellow boxes indicate repeat regions. **B**) Amino acid sequence alignment of the PHIST domains from PBANKA_114540 and PBANKA_122900. Tryptophan residues are shaded in gray. The predicted helical segments are shown above the alignment, marked by “H”. Below the alignment, stars indicate identical aa residues, 2 dots indicate conserved substitutions and one dot indicates semi-conserved substitutions. **C**) Transcript expression analysis of the *phist* genes during the *P*. *berghei* life cycle. Transcript levels for *PBANKA_114540* and *PBANKA_122900* throughout the parasite life cycle was analyzed by real-time quantitative RT-PCR using cDNA that was prepared from synchronized asexual stages, gametocytes and mosquito stage parasites. Transcript expression was normalized to the expression of the control gene *hsp70*. *Pbama-1* was used as a stage-specific control for schizont transcription; *PbCCp1* for gametocytes; and *PbCSP* for oocysts and sporozoites. 5 h, 12 h, 16 h, 20 h and 23 h indicate hours after injecting synchronized schizonts into the tail vein of a mouse. SC, purified schizonts; G, gametocytes; 5 d, 5-day oocysts; 8 d, 8-day oocysts; 10 d, 10-day oocysts; 15 d, 15-day oocysts; SG, salivary gland sporozoites. The composition of the asexual population with respect to life cycle stage is shown for each time point in **D**), expressed as percentages of the total population.

The *P*. *berghei* PHIST proteins PBANKA_114540 and PBANKA_122900 possess similar features to *P*. *falciparum* PHIST proteins; namely, signal peptides and PEXEL/HT trafficking motifs; and single PHIST domains, which are divergent in aa sequence with respect to each other (Fig 5A and 5B). Within the single predicted ORF of *PBANKA_070080* we were unable to identify a signal peptide sequence, and there were no attractive upstream ORFs suggestive of an erroneous gene model. We thus propose that *PBANKA_070080* is a pseudogene. The remaining P. *berghei phist* genes possess the typical 2-exon gene structure, in which the signal peptide is encoded on the first exon and the PEXEL/HT motif is located within the second exon (Fig 5A). To determine the transcript levels of the *P*. *berghei phist* genes throughout the parasite life cycle, quantitative real-time RT-PCR was performed using cDNA that was prepared from synchronized asexual stages, gametocytes and mosquito stage parasites (Fig 5C and 5D). *PBANKA_114540* and *PBANKA_122900* are predominantly transcribed in mature schizont stage parasites, and the transcript levels in schizonts were comparable to the levels of *ama1* transcripts (*PBANKA_091500*), which was used as a positive marker for schizont stage transcription in addition to the control housekeeping gene, *hsp70* (*PBANKA_091441*). Low expression of *PBANKA_114540* was observed in gametocytes and no transcripts were detected in mosquito stages. Lack of detectable *PBANKA_070080* transcripts supports the above annotation as a pseudogene, and accordingly our further analyses of *phist* genes did not include *PBANKA_070080*.

### Cellular localization of *P*. *berghei* PHIST proteins

**Fig 6:**
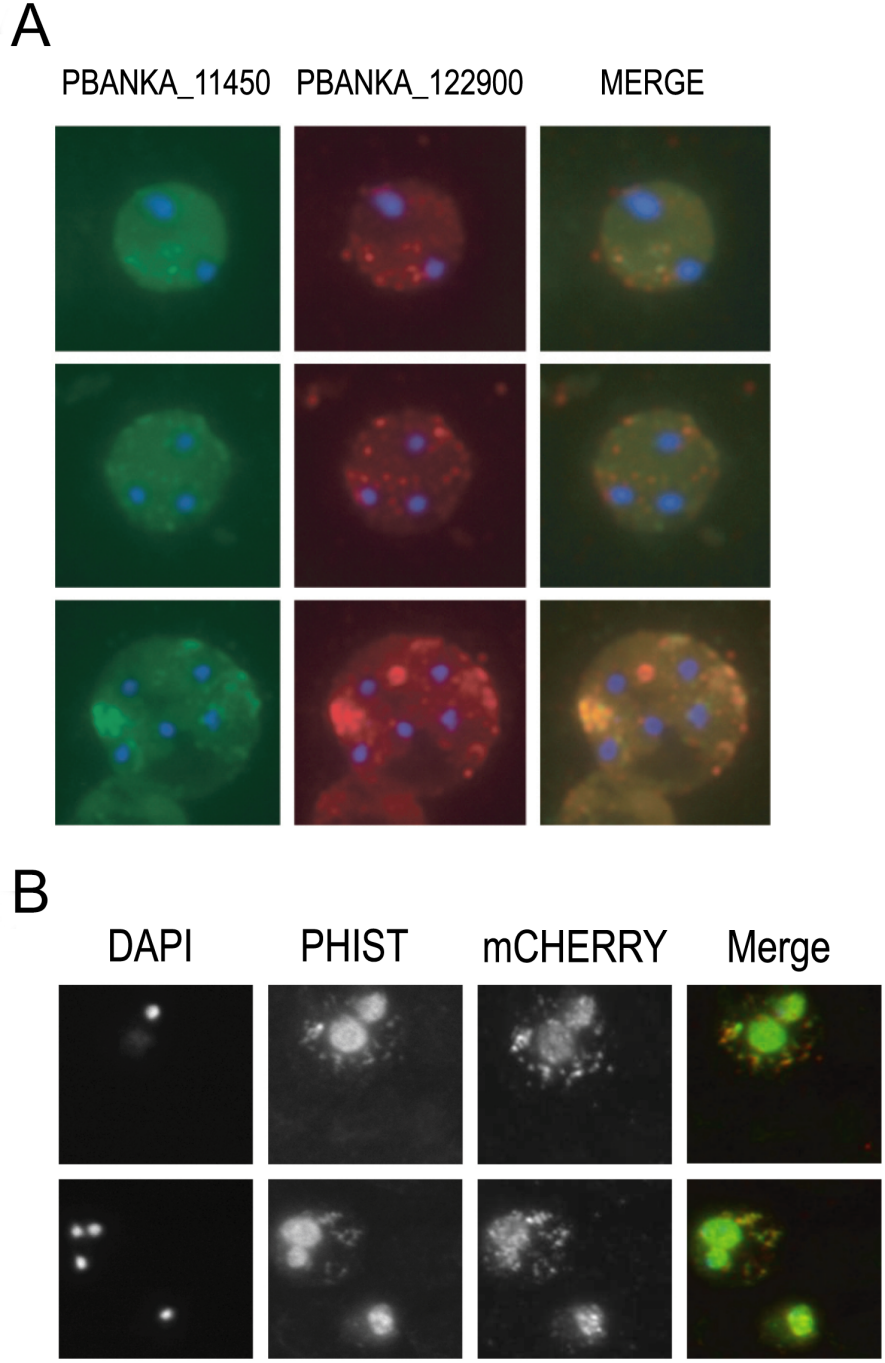
Immunolocalization of *P*. *berghei* PHIST proteins PBANKA_114540, PBANKA_122900, and IBIS within infected erythrocytes. **A**) PBANKA_114540 co-localizes with PBANKA_122900 within erythrocyte cytoplasmic vesicles. Co-localization was assayed using rabbit polyclonal anti-PBANKA_114540 followed by goat anti-rabbit IgG conjugated with Alexa 488 (green), followed by rabbit polyclonal anti-PBANKA_122900 followed by goat anti-rabbit Alexa 595 (red). Nuclei were stained with DAPI (blue). Control experiments using secondary antibodies were negative. **B**) Co-localization of mCherry-tagged IBIS protein PBANKA_136550 with the PHIST protein PBANKA_122900 in fixed erythrocytes, by staining with rabbit polyclonal anti-PBANKA_122900 followed by goat anti-rabbit IgG conjugated with Alexa 488. Parasite nuclei were stained with Hoechst (blue). Both proteins partially co-localize inside vesicles in the erythrocyte cytoplasm.

Immunolocalization studies using affinity purified rabbit polyclonal anti-sera against PBANKA_114540 and PBANKA_122900 revealed that both proteins are exported to the erythrocyte cytoplasm and observed throughout the intraerythrocytic life cycle (Fig 6A). In early asexual stages, both proteins exhibited a punctate, vesicle-like localization in the erythrocyte cytoplasm while in more mature stages the protein distribution appeared more diffuse. Immunofluorescence analysis also suggests that PBANKA_114540 and PBANKA_122900 associate with vesicle-like structures, or aggregations, in the erythrocyte cytoplasm (Fig 6A). In addition, the proteins co-localize, suggesting they share a common export system, or aggregation, in the rodent malaria parasites (Fig 6A). Staining of non-permeabilized cells was negative, indicating that PBANKA_114540 and PBANKA_122900 are not exposed on the surface of the infected erythrocyte (data not shown). We did not observe association of either PHIST protein with the erythrocyte membrane.

To compare the punctate localization pattern of PHIST domain proteins with other *P*. *berghei* exported proteins that exhibit similarly punctate patterns, and to address possible co-localization within a single vesicle transport system, we analyzed the co-localization of PBANKA_136550 (IBIS1; [46]) and the PHIST protein PBANKA_122900 by IFA. We utilized a transgenic parasite line that expresses a mCherry-tagged version of PBANKA_136550. Analysis of live infected erythrocytes showed export of mCherry-PBANKA_136550 into the cytoplasm of the erythrocyte and localization in punctate vesicle-like structures (Fig 6B), as described for this protein by Ingmundson *et al*. [46]. Staining of mCherry-PBANKA_136550 parasites with anti-TER antibodies, a marker of the erythrocyte surface membrane, showed that PBANKA_136550 localizes exclusively within the erythrocyte cytoplasm (data not shown). Staining of fixed erythrocytes infected with mCherry-PBANKA_136550 parasites with anti-PBANKA_122900 antibodies showed a significant overlap between the fluorescence signals, indicating that both proteins localize within the same erythrocyte compartment (Fig 6B).

### Functional analysis of *P*. *berghei* PHIST by targeted gene deletion

To address the function of PBANKA_114540 and PBANKA_122900 in *P*. *berghei*, we attempted to disrupt the genes via double-cross-over homologous recombination (S4 and S5A Figs, respectively). The *PBANKA_114540* disruption construct was designed using the pDEF-hDHFR vector, while the *PBANKA_122900* disruption construct used the vector b3D.DT^H.^D. The disruption vectors were transfected into *P*. *berghei* ANKA schizonts and transformants were selected via pyrimethamine drug pressure. In 3 independent transfection experiments we were unable to select mutant parasites with a disrupted *PBANKA_114540* locus, suggesting that this gene is essential in asexual stage parasites. In contrast, deletion of *PBANKA_122900* was achieved in a parallel experiment; as well as disruption of the *PbTLP* locus as a control, and correct disruption of these genes was verified by diagnostic PCR and Southern blot analysis (S5B and S5C Fig). Two *PBANKA_122900* disruptant clones were selected for further characterization. Transcripts of *PBANKA_122900* were not detected by RT-PCR in asexual stages of disruptant parasites (S5D Fig), confirming a successful deletion of the locus. The ability to generate disruptant parasites demonstrated that PBANKA_122900, as opposed to PBANKA_114540, is not essential for asexual intraerythrocytic development.

The *PBANKA_122900* KO parasite lines showed normal gametocyte and ookinete formation in vitro (data not shown). Following transmission to mosquitoes the KO parasite lines produced oocysts that were morphologically indistinguishable from the wt control (S4 Table), and formed normal numbers of salivary gland sporozoites (S5 Table). Sporozoites from KO lines showed wt levels of hepatocyte invasion and developed into mature exoerythrocytic forms similarly to wt (S6 Fig). In addition, the infectivity of KO sporozoites *in vivo* was similar to wt, as shown by prepatent period after intravenous injection of 10^2^ to 10^3^ sporozoites (S6 Table). Taken together, the assays employed in this study could not discern a role for PBANKA_122900 during development in the mosquito or the liver.

**Fig 7:**
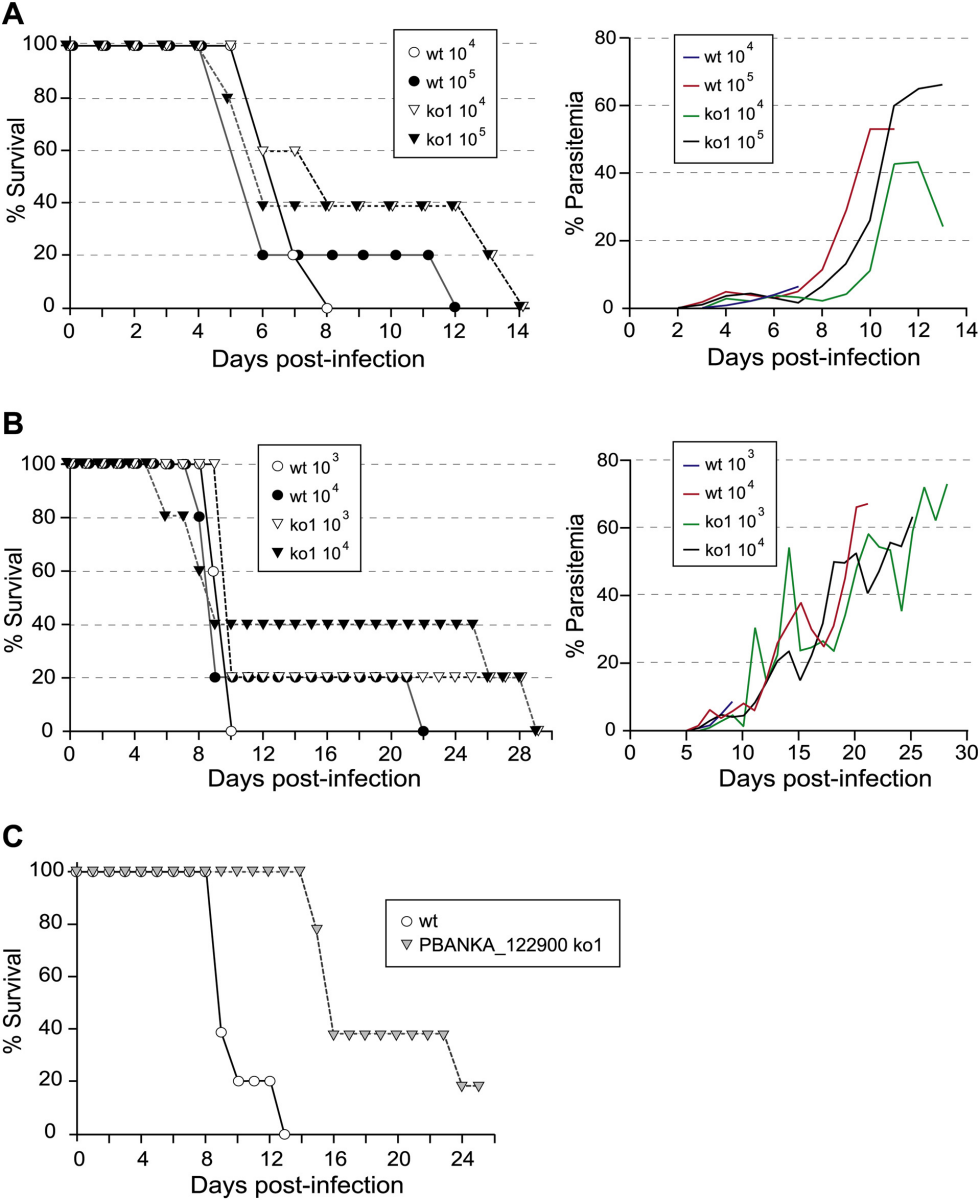
Dynamics of malaria infection after inoculation with erythrocytic stage parasites and sporozoites. Survival curves and daily parasitemia of **A)** Swiss Webster or **B)** C57Bl/6 mice injected intravenously with the indicated number of schizonts or mixed asexual stages of *P*. *berghei* ANKA wt or PBANKA_122900 KO, respectively. Each group contained 5 mice. The average parasitemia for all mice in each group is plotted. C) Survival curves of Swiss Webster mice injected intravenously with 1,000 sporozoites of *P*. *berghei* ANKA wt or PBANKA_122900 KO. Mice were monitored daily and the percentage surviving on each day is plotted. There were 5 mice per group and this experiment was repeated with similar results.

To describe the development of the asexual blood stages of *PBANKA_122900* KO lines we determined the course of infection in Swiss Webster and C57Bl/6 mice following either inoculation with sporozoites or intraerythrocytic stages. An apparent trend towards long survival was observed in Swiss Webster mice (Fig 7A) or C57Bl/6 mice (Fig 7B) that were intravenously (i.v.) injected with 10^4^ and 10^5^ schizonts (Swiss Webster mice) or 10^3^ or 10^4^ mixed asexual stages (C57Bl/6 mice) of *PBANKA_122900* KO parasites. However, the trend does not have statistical support. When we monitored the survival of mice after i.v. inoculation of 1,000 *P*. *berghei* ANKA wt or *PBANKA_122900* k KO sporozoites, we observed a longer survival time of mice infected with *PBANKA_122900* KO than those infected with wt parasites (Fig 7C), which is in support of the above mild growth delay of asexual blood stages. We did not detect differences in reticulocyte versus normocyte preference or a lower production of merozoites per schizonts; specifically, analysis of the percentage of young and mature erythrocytes infected with wt or KO parasites, at between 1 and 3% parasitemia before mice started producing reticulocytes, did not reveal a preference for invasion of one cell type by KO parasites (data not shown). In addition, erythrocytes infected with wt or KO parasites exhibited the same average number of merozoites per schizont (data not shown). In conclusion, further analysis of growth characteristics is required to discern any effects of disruption of PBANKA_122900 on the growth of asexual blood stages and lethality of KO parasites.

## Discussion

A major determinant of the pathogenicity of malaria parasites is their ability to remodel infected erythrocytes, in order to facilitate parasite metabolism, intraerythrocytic development and evasion from the host immune responses. The process of erythrocyte remodeling is poorly understood, but requires a complex parasite-derived trafficking network that exports an extensive repertoire of *Plasmodium* proteins to the erythrocyte cytoplasm or surface ([16, 17]; reviewed in [47–49]. In *P*. *falciparum* 65 genes encode PHIST proteins and 7 genes encode *resa-like* proteins. Unlike the *P*. *falciparum var* and *rif* gene families, for which expansion is thought to have been driven in large part by immune pressure, amplification of *phist* genes appears to have derived from other pressures, such as selection for functional diversity and stage-specific expression. It is also possible that the PHIST domains mediate a single function, or closely related functions, and amplification was driven by a requirement for exceedingly high amounts of exported PHIST protein. In this model it might be proposed that many gene loci are required to generate sufficient protein, for an unknown role within the erythrocyte cytoplasm, and that gene amplification was perhaps aided by the subtelomeric localization of *phist* genes within neighborhoods of other highly amplified genes. Subsequent divergence of the loci might have been required to counter chromosomal instability caused by many copies of homologous sequences. Whatever the model explaining *phist* gene amplification, it must be reconciled that only 2 functional *phist* genes are present in the genomes of rodent malaria parasites, in contrast to the great expansion found in *P*. *falciparum*, *P*. *vivax* and *P*. *knowlesi*. Moreover, *resa-like* genes have been thus far observed only in *P*. *falciparum*, and are absent in other *Plasmodium* species, and comparative models of erythrocyte modification should explain a species-specific function for RESA-like proteins.

*Phist* and *resa-like* transcripts predominate during the intraerythrocytic asexual stages of *P*. *falciparum*, with expression of a subset of genes observed in gametocytes [14]. In addition, transcripts span a range of abundance and stage-specific expression, perhaps supporting a hypothesis of functional diversification of PHIST proteins. Transcripts for the *P*. *berghei phist* genes *PBANKA_114540* and *PBANKA_122900* were also detected in intraerythrocytic stages. Many *P*. *falciparum* PHIST and RESA-like proteins have been observed to be non-essential *in vitro* as well as *in vivo*. Deletion of the left arm of chromosome 2, which contains the *resa-like* gene *PFB0085c*, has been described in fresh field isolates as well as in laboratory strains, such as Dd2 [9, 50]. *PFA0110w* (*resa*) has been shown to be dispensable for *in vitro* growth, as indicated by 2 independent gene knockout studies [51, 20]. A gene disruption study described the successful knockout of 13 *phist* and *resa-like* genes [20]. Although studies reported the non-essentiality of PHIST and RESA-like proteins *in vitro*, it is possible that the breadth of the repertoire insures redundant, compensatory functions. Our attempts to disrupt the *P*. *berghei phist* genes yielded disruption of *PBANKA_122900* but not *PBANKA_114540* in multiple attempts, demonstrating that while the former is non-essential, the latter is likely critical for asexual parasite growth.

The function of PHIST and RESA-like proteins remains unknown. RESA was shown to bind to the erythrocyte cytoskeletal protein spectrin, and this binding leads to an increased mechanical stability of the cell membrane, which reduces the cell’s susceptibility to heat-induced vesiculation and increases resistance to multiple parasite invasion [52]. It is thus proposed that RESA guards the erythrocyte against thermal damage during febrile episodes, and prevents multiple parasitism. Loss of the *phist* gene *PFD1170c* resulted in knobless parasites in which a defect in transfer of KAHRP from Maurer’s clefts to the erythrocyte membrane was observed, suggesting a function for this protein in knob formation [20]. Disruption of the *phist* gene *MAL7P1*.*172* ablated expression of PfEMP1 on the erythrocyte surface, although the protein was detected in Maurer’s clefts, suggesting a role in trafficking and display of PfEMP1 [20]. Knockout of *resa*, the *resa-like* gene *PFB0920w* and the *phist* gene *PF14_0018* resulted in altered cell rigidity [20]. Deletion of the PHISTc gene *LyMP* (PFE1605w; [25]) reduced adhesion of infected erythrocytes to CD36 by 55%. However, display of PfEMP1 on the erythrocyte surface and formation of knobs were not affected, suggesting that LyMP modulates cytoadhesion by a mechanism independent of any identified previously. In addition, a recent study demonstrated that the ATS domain of PfEMP1 associates with the PHIST protein PFI1780w [21], as well as the binding of a PHIST to band 4.1 [22]. Together, these data suggest that the *P*. *falciparum* PHIST and RESA-like proteins underpin multiple functions in the process of erythrocyte remodeling essential for parasite survival, multiplication and evasion of the host immune system. Our studies on the disruption of the *P*. *berghei phist* gene *PBANKA_122900* did not yield a clear phenotype that allowed us to propose a specific function to this protein.

The erythrocytic trafficking of the *P*. *falciparum* and *P*. *berghei phist* proteins characterized in this study confirmed the prediction of targeting based upon the presence of a PEXEL/HT motif. No association with the erythrocyte surface was observed, suggesting a final cellular destination and function within the erythrocyte cytoplasm. The epitope-tagged *P*. *falciparum* PHIST proteins, PFE1600w and PFE1605w, were observed exported to the erythrocyte cytoplasm and in partial co-localization with a marker of Maurer’s clefts, SBP1. Our data is in accordance with a recent study on PFE1605w (LyMP) that shows a punctate fluorescence in the cytosol of erythrocytes infected with late stage parasites, using anti-LyMP antibodies or HA-tagged endogenous protein [25]. The authors show by immunoelectron microscopy that LyMP (PFE1605w) localizes in distinct puncta at the erythrocyte membrane, in the region between the knobs. Solubility assays suggested that LyMP interacts with the erythrocyte membrane skeleton, and this was confirmed by showing that the C-terminus of LyMP binds to inside-out vesicles *in vitro* [25]. The *P*. *berghei* PHIST proteins, PBANKA_114540 and PBANKA_122900, were also observed exported to the erythrocyte cytoplasm and exhibited a co-localization within punctate, vesicle-like structures that resembled *P*. *falciparum* Maurer’s clefts. Moreover, the PHIST protein PBANKA_122900 co-localized with PBANKA_136550 (IBIS), a *P*. *berghei* exported protein that exhibits a similar punctate pattern, suggesting localization within a common vesicle transport system. The physiological function of the structures containing the PHIST proteins of *P*. *berghei* in infected erythrocytes, as well as their cargo, remains unknown. However, their presence supports the burgeoning idea that many dynamic vesicular trafficking pathways exist in the host cytoplasm; for example, to mediate the transport of proteins/lipids from the host plasma membrane to the PVM or vice-versa.

Ultrastructural studies of intraerythrocytic stages have predominantly focused on *P*. *falciparum*-infected erythrocytes and describe a membranous system in the cytoplasm of the host cell; including Maurer’s clefts, flattened cisternae-like structures that carry several characterized exported proteins [53, 54]; J-dots which contain HSP40 [55]; tethers, electron dense tubular structures that connect Maurer’s clefts to other membranes [56, 57]; and additional membrane populations of unknown function [58]. Several studies have described vesicle-like structures in the cytoplasm of *P*. *berghei*-infected erythrocytes [46, 58–60], but these structures have not been correlated to those described for *P*. *falciparum*. Akinyi *et al*. [24] propose that PBANKA_122900 is orthologous to the *P*. *vivax* and *P*. *cynomolgi* PHIST domain proteins, *pvphist/cvc-81*_*95*_ and *pcyphist/cvc-81*_*95*_, respectively, and the orthology is supported by reciprocal BLAST analyses and gene synteny. Efforts to disrupt *pcyphist/cvc-81*_*95*_ were unsuccessful, suggesting that this protein is essential in *P*. *cynomolgi*. If the orthologous (vertical) relationship holds true, as well as the essentiality of the *P*. *cynomolgi* version, then it would suggest that either the orthologous PHIST domains have different functions in the two pathogens, or that the conserved function is essential only in *P*. *cynomolgi* and not rodent malaria parasites. In the elegant immunoelectron tomography aspect of the study the authors showed that *P*. *vivax* and *P*. *cynomolgi* PHIST/CVC-81_95_ localizes to the cytoplasmic face of the caveola-vesicle complexes (CVCs); specifically, indentations of the infected erythrocyte membrane that associate with vesicles that are suggested to function in nutrient transport or release of parasite metabolites from infected erythrocytes [24]. This localization might help to instruct models of PHIST domain function, because CVCs, also termed Schüffner's dots, are specific to *P*. *vivax* and *P*. *cynomolgi*, whereas other *Plasmodium* species lack CVCs but retain Maurer’s clefts.

In *P*. *berghei* infected erythrocytes 7 parasite proteins have been characterized to date that are exported beyond the PVM, and share a punctate, vesicle-like localization in the cytoplasm of infected erythrocytes, namely: small exported proteins 2 (SEP2) and 3 (SEP3); schizont membrane-associated cytoadherence protein (SMAC) and PBANKA_136550 (also termed IBIS1); the 2TM protein, PbCP1; and the 2 PHIST proteins ([38, 46, 59, 60]; and this manuscript]. Five of these proteins contain a PEXEL/VTS motif while SEP2 and 3 do not. SEP 2 and 3 co-localize within the same vesicular structures, whereas the PHIST proteins co-localize with PBANKA_136550, but it’s unknown if SEP2 and 3 also co-localize with PHIST and PBANKA_136550. Parasite-derived structures have been recently described within erythrocytes infected with rodent malaria parasite [61, 62], but to date the PHIST domain, which is exported and displays vesicular localization in both *P*. *falciparum* and *P*. *berghei* infected erythrocytes, is the only marker in support of an analogy to Maurer’s clefts. Our data supports that *P*. *berghei* is a facile model system for *in vivo* studies of protein export and host-parasite interactions in intraerythrocytic asexual stages.

## Supporting Information

S1 Fig Protein domain architectures for 65 *P*. *falciparum* PHIST-domain encoding genes. The ruler above the architectures indicates protein lengths in aa, drawn to scale. Blue regions represent signal sequences, often times recessed from the start methionine; red regions represent the PEXEL/HT motif; and green regions represent the PHIST domain. The inset lists predicted pseudogenes. Yellow and orange highlight indicate genes whose transcript expression profile was assayed by real-time RT-PCR. Orange highlight show the genes that were epitope-tagged for protein expression and localization studies. Designations A, B and C refer to PHIST types described in Sargeant et al. [12]. “g’cyte” indicates gametocytes. The *phist* genes were identified by the following methods: i) iterative PSI-BLAST screening of *P*. *falciparum* 3D7 isolate information in GenBank using a variety of PHIST domains as aa queries; ii) genome walking using Artemis to identify all predicted ORFs within 150 Kbp of each telomere of all chromosomal arms of the *P*. *falciparum* 3D7 isolate, followed by annotation by BLAST analysis using these ORFs as queries of GenBank, as well as passing through the web-based NCBI Conserved Domain database (expect value set at 10; http://www.ncbi.nlm.nih.gov/Structure/cdd/wrpsb.cgi); and iii) analysis of flanking regions of predicted *phist* genes which lack predicted signal peptides and PEXEL/HT trafficking motifs.
(TIF)

S2 Fig Transcript levels at time points throughout the asexual intraerythrocytic lifecycle for the *P*. *falciparum* isolate 3D7 for 11 *phist* genes A) and 7 *resa-like* genes B). Microarray transcript level data for each gene was collected from PlasmoDB (www.plasmodb.org); and further information is available from [63]. The expression profiles for the *phist* genes appear to cluster throughout the asexual lifecycle, as indicated for two genes highlighted in red and blue **A**). Expression data is not shown in **A)** for two *phist* genes described in Fig 2, PFE1600w and PFI1770w, because their expression data in Plasmodb is derived from Le Roch et al [64] and is presented in a different format.
(TIF)

S3 Fig Characterization of *P*. *falciparum* parasite lines expressing c-myc epitope tagged PHIST proteins. **A**). Schematic representation of the expression vector, designed to insert 3 tandem c-myc epitopes at the carboxy-terminus of PFE1600w or PFE1605w. Expression of transgenes is driven by the *P*. *falciparum hrp3* promoter, and terminated by the 3’UTR of *P*. *falciparum hrp2*. **B**). Plasmid copy number in *P*. *falciparum* stably transformed with constructs encoding *PFE1600w* or *PFE1605w*. The copy numbers of the c-myc-tagged genes *PFE1600w* and *PFE1605w* were determined by quantitative real time PCR. Values were normalized to the single copy gene *arginyl-tRNA synthetase* (*PFL0900c*). Values represent the average of 2 independent experiments, and error bars indicate the standard deviation. **C**). Quantification of episomal gene expression of c-myc-tagged *PFE1600w* and *PFE1605w*. Transcript levels of endogenous and episomal genes were analyzed by quantitative real time PCR using cDNA prepared from *P*. *falciparum* stably transformed with constructs encoding *PFE1600w* or *PFE1605w*. Values were normalized to the expression of the control gene *arginyl-tRNA synthetase* (*PFL0900c*). Gray bars indicate the endogenous gene expression and white bars represent the episomal gene expression. Synchronized parasites were harvested at different time points after Percoll-sorbitol purification: 3 h, recently invading parasites; 6 h, early ring stage; 12 h, mid-ring stage, 22 h, late ring stage; 30 h, early-trophozoite stage; 42 h, mid-trophozoite stage; 48 h, late trophozoite stage; SC, schizont stage. **D**). Western blot with anti-c-myc monoclonal antibodies to detect c-myc-tagged PFE1600w and PFE1605w, of approximate molecular weights 64 kDa and 65 kDa, respectively. A parasite line that does not express the c-myc epitope but is transformed with the same plasmid vector was used as a negative control. Anti-Pf39 serum was used as a positive control for protein loading.
(TIF)

S4 Fig Targeted gene disruption of *PBANKA_114540* in *P*. *berghei* ANKA via double homologous recombination. Schematic of the *PBANKA_114540* locus, disruption plasmid and expected disrupted locus. Thick black lines indicate the 5’- and 3’-UTRs of *PBANKA_114540* and boxes indicate the coding sequence; gray boxes indicate the regions of *PBANKA_114540* that were used to target homologous recombination within the disruption plasmid; *hdhfr* indicates the coding sequence of the selectable marker, flanked by thin lines representing the 5’- and 3’-UTRs. Double diagonal lines interrupting the 5’- and 3’-UTRs of the *hdhfr* cassette indicate that these regions are not in scale. Letters “a” to “d” indicate the locations of PCR primers that yield products diagnostic of wt and disrupted loci.
(TIF)

S5 Fig Targeted gene disruption of *PBANKA_122900* in *P*. *berghei* ANKA, via double homologous recombination. **A**) Schematic of the *PBANKA_122900* locus, disruption plasmid and expected disrupted locus. Thick black lines indicate the 5’- and 3’-UTRs of *PBANKA_122900* and boxes indicate the coding sequence; gray boxes indicate the regions of *PBANKA_122900* that were used to target homologous recombination within the disruption plasmid; *hdhfr* indicates the coding sequence of the selectable marker, flanked by thin lines representing the 5’- and 3’-UTRs. Double diagonal lines interrupting the 5’- and 3’-UTRs of the *hdhfr* cassette indicate that these regions are not in scale. Letters “a” to “d” indicate the locations of PCR primers that yield products diagnostic of wt and disrupted loci; and “e” to “h” indicate the location of primers used in the RT-PCR. The probe used to screen the Southern blot is indicated as a solid horizontal bar above one of the gray boxes. Abbreviations are C, *Cla*I; and E, *EcoR*I. **B**) EtBr-stained agarose gel electrophoresis of PCR products amplified from primer pairs diagnostic of wt locus and integration events. Results from 2 disruptant clones are shown; ko1 and ko2. Different lanes show results with the indicated primer sets which are shown in panel A. **C**) Southern blot of *Cla*I and *Eco*RI digested genomic DNA from wt and disruptant lines showing complete disruption of the *PBANKA_122900* gene locus. **D**) RT-PCR with primer set “ef” shows loss of *PBANKA_122900* expression in disruptant parasites. Primer set “gh” shows the presence of the drug selectable marker cassette in transgenic parasites. Reverse transcriptase (RT) and sham controls are indicated above the lanes by “+” and “-”, respectively. The *P*. *berghei* gene, *ama-1* (*PB000821*.*01*.*0*), was used as a positive control (data not shown).
(TIF)

S6 Fig Infectivity of *PBANKA_122900* KO to hepatocytes *in vitro*. *P*. *berghei* wt and 2 clones of *PBANKA_122900* KO sporozoites (ko1 and ko2) were added to Hepa 1–6 cells for 1 h and either fixed for invasion assay (grey bars) or grown for an additional 2 days before fixing and staining for exoerythrocytic forms (EEFs, black bars). Fifty and 20 fields per well at 400x magnification were counted for invasion and development assays, respectively. Shown are the means ± SD of triplicates.
(TIF)

S1 Table Specific primer sets used to amplify by real time RT-PCR the members of the *phist* and *resa-like* gene familes, as well as control genes. (DOCX)

S2 Table Primer sets used to amplify fragments cloned in epitope-tagged constructs, knockout contructs and diagnostic PCR of disrupted locus. (DOCX)

S3 Table Revised annotations for Sargeant et al. [12]. (DOCX)

S4 Table Oocyst development of PBANKA_122900 KO populations in the mosquito midgut. (DOCX)

S5 Table Development of PBANKA_122900 KO sporozoites in the mosquito. (DOCX)

S6 Table Infectivity of PBANKA_122900 knockout sporozoites as determined by prepatent period. (DOCX)
