# Supplementary figures and images for: The Plasmodium PHIST and RESA-Like Protein Families of Human and Rodent Malaria Parasites
Source: PLoS One. 2016 Mar 29;11(3):e0152510. doi: 10.1371/journal.pone.0152510 (PMC4811531; doi:10.1371/journal.pone.0152510)

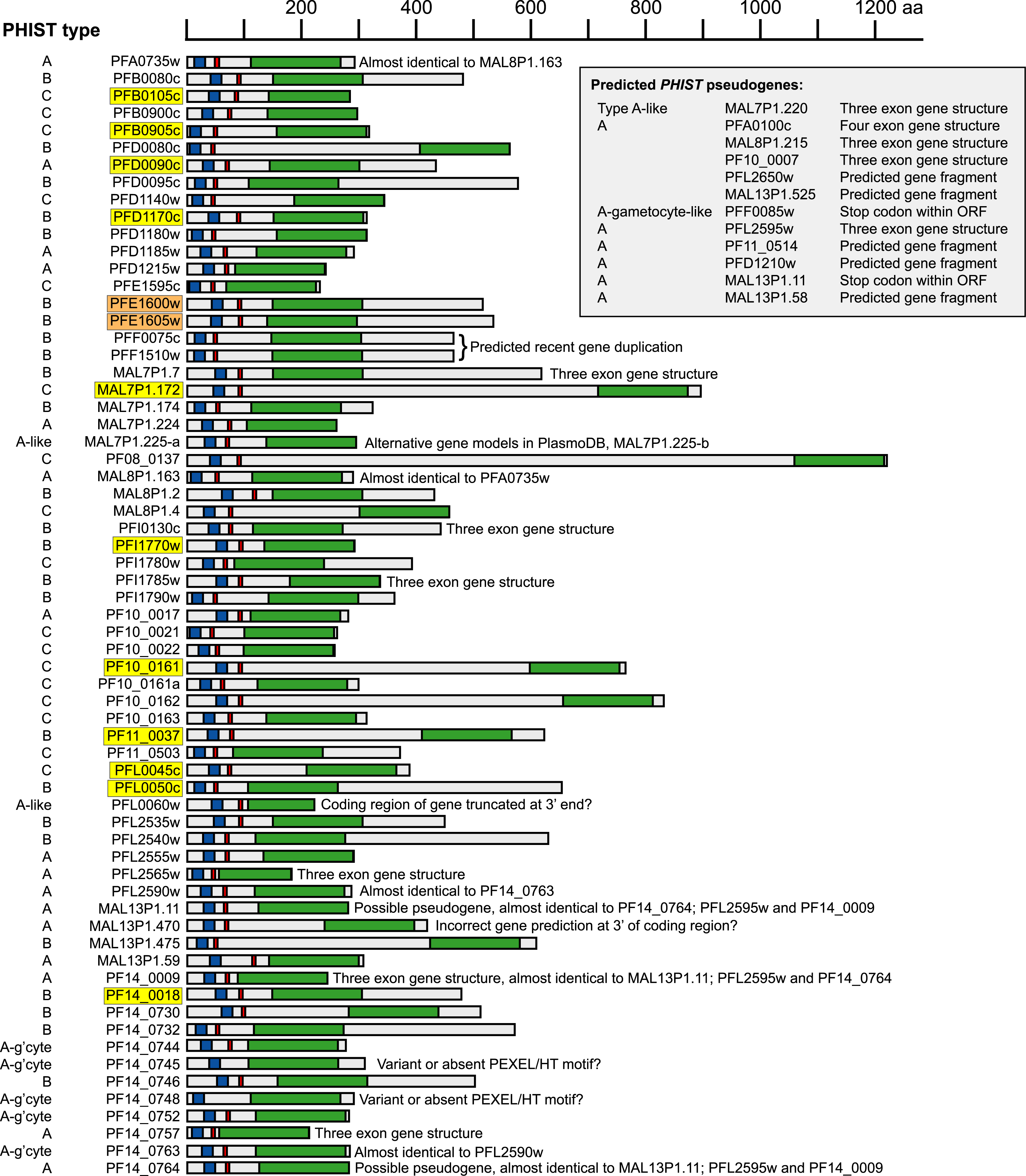

Supplement: S1 Fig — The ruler above the architectures indicates protein lengths in aa, drawn to scale. Blue regions represent signal sequences, often times recessed from the start methionine; red regions represent the PEXEL/HT motif; and green regions represent the PHIST domain. The inset lists predicted pseudogenes. Yellow and orange highlight indicate genes whose transcript expression profile was assayed by real-time RT-PCR. Orange highlight show the genes that were epitope-tagged for protein expression and localization studies. Designations A, B and C refer to PHIST types described in Sargeant et al. [12]. “g’cyte” indicates gametocytes. The phist genes were identified by the following methods: i) iterative PSI-BLAST screening of P. falciparum 3D7 isolate information in GenBank using a variety of PHIST domains as aa queries; ii) genome walking using Artemis to identify all predicted ORFs within 150 Kbp of each telomere of all chromosomal arms of the P. falciparum 3D7 isolate, followed by annotation by BLAST analysis using these ORFs as queries of GenBank, as well as passing through the web-based NCBI Conserved Domain database (expect value set at 10; http://www.ncbi.nlm.nih.gov/Structure/cdd/wrpsb.cgi); and iii) analysis of flanking regions of predicted phist genes which lack predicted signal peptides and PEXEL/HT trafficking motifs. (TIF) [file pone.0152510.s001.tif]

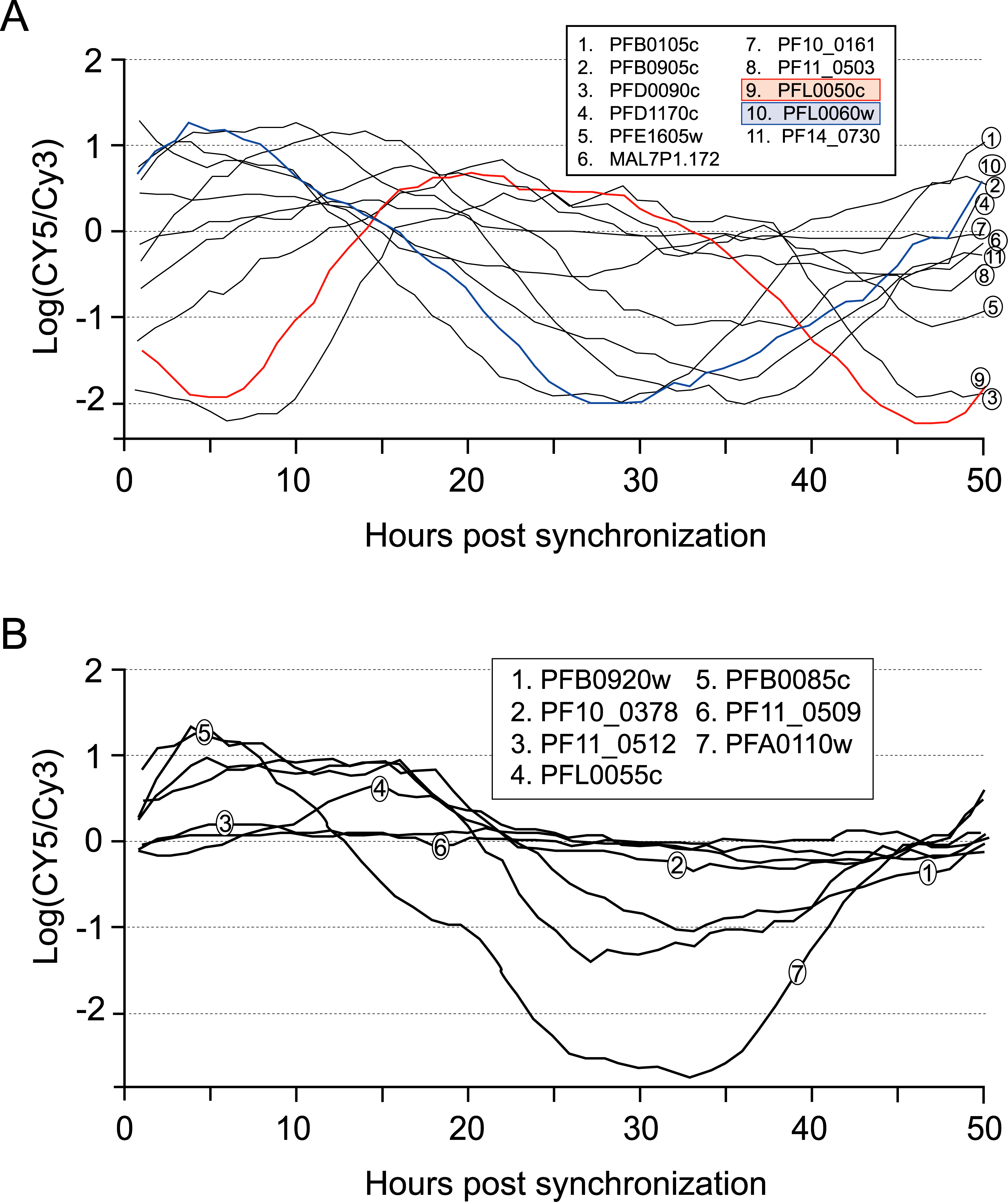

Supplement: S2 Fig — Microarray transcript level data for each gene was collected from PlasmoDB (www.plasmodb.org); and further information is available from [63]. The expression profiles for the phist genes appear to cluster throughout the asexual lifecycle, as indicated for two genes highlighted in red and blue A). Expression data is not shown in A) for two phist genes described in Fig 2, PFE1600w and PFI1770w, because their expression data in Plasmodb is derived from Le Roch et al [64] and is presented in a different format. (TIF) [file pone.0152510.s002.tif]

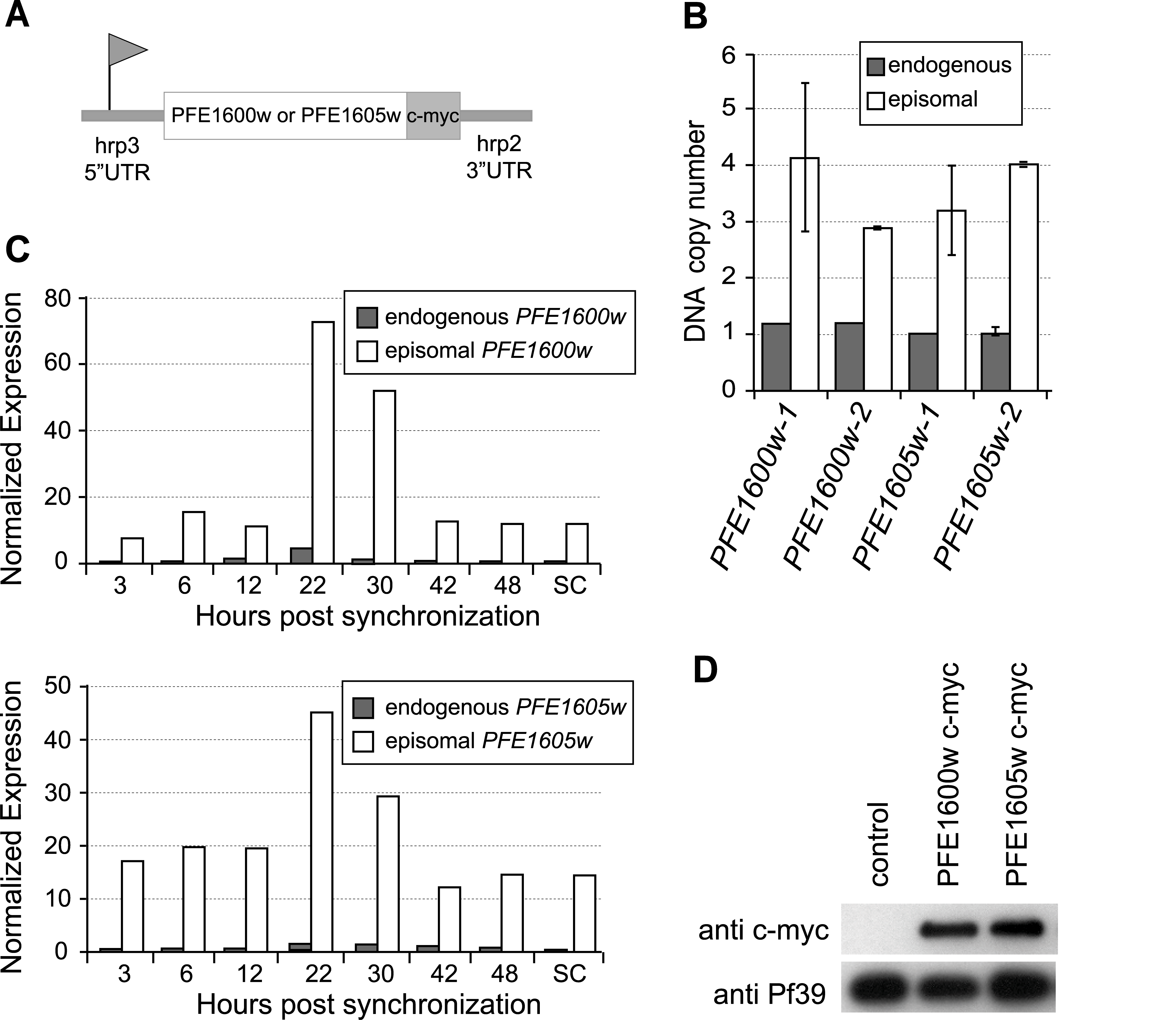

Supplement: S3 Fig — A). Schematic representation of the expression vector, designed to insert 3 tandem c-myc epitopes at the carboxy-terminus of PFE1600w or PFE1605w. Expression of transgenes is driven by the P. falciparum hrp3 promoter, and terminated by the 3’UTR of P. falciparum hrp2. B). Plasmid copy number in P. falciparum stably transformed with constructs encoding PFE1600w or PFE1605w. The copy numbers of the c-myc-tagged genes PFE1600w and PFE1605w were determined by quantitative real time PCR. Values were normalized to the single copy gene arginyl-tRNA synthetase (PFL0900c). Values represent the average of 2 independent experiments, and error bars indicate the standard deviation. C). Quantification of episomal gene expression of c-myc-tagged PFE1600w and PFE1605w. Transcript levels of endogenous and episomal genes were analyzed by quantitative real time PCR using cDNA prepared from P. falciparum stably transformed with constructs encoding PFE1600w or PFE1605w. Values were normalized to the expression of the control gene arginyl-tRNA synthetase (PFL0900c). Gray bars indicate the endogenous gene expression and white bars represent the episomal gene expression. Synchronized parasites were harvested at different time points after Percoll-sorbitol purification: 3 h, recently invading parasites; 6 h, early ring stage; 12 h, mid-ring stage, 22 h, late ring stage; 30 h, early-trophozoite stage; 42 h, mid-trophozoite stage; 48 h, late trophozoite stage; SC, schizont stage. D). Western blot with anti-c-myc monoclonal antibodies to detect c-myc-tagged PFE1600w and PFE1605w, of approximate molecular weights 64 kDa and 65 kDa, respectively. A parasite line that does not express the c-myc epitope but is transformed with the same plasmid vector was used as a negative control. Anti-Pf39 serum was used as a positive control for protein loading. (TIF) [file pone.0152510.s003.tif]

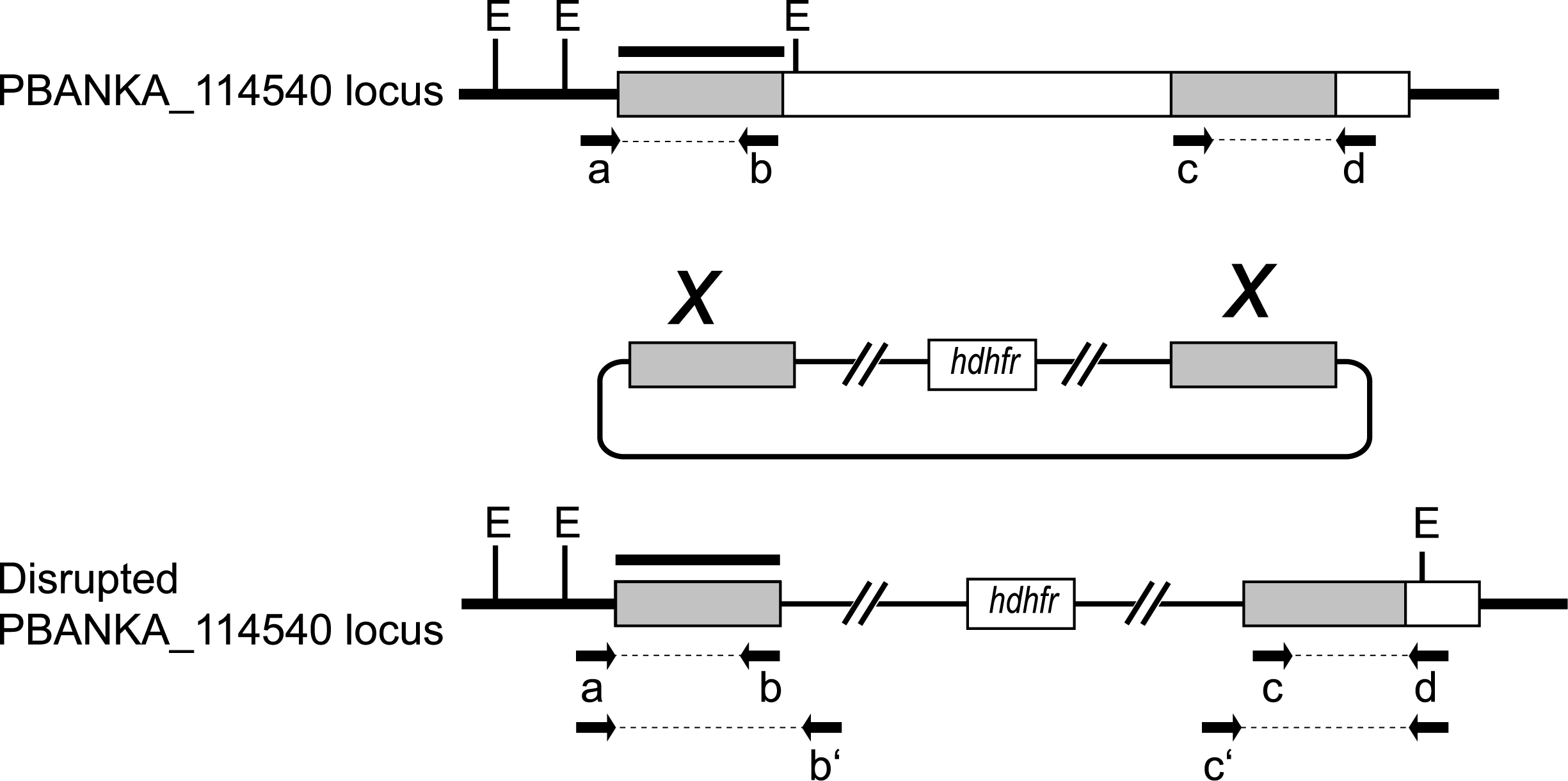

Supplement: S4 Fig — Schematic of the PBANKA_114540 locus, disruption plasmid and expected disrupted locus. Thick black lines indicate the 5’- and 3’-UTRs of PBANKA_114540 and boxes indicate the coding sequence; gray boxes indicate the regions of PBANKA_114540 that were used to target homologous recombination within the disruption plasmid; hdhfr indicates the coding sequence of the selectable marker, flanked by thin lines representing the 5’- and 3’-UTRs. Double diagonal lines interrupting the 5’- and 3’-UTRs of the hdhfr cassette indicate that these regions are not in scale. Letters “a” to “d” indicate the locations of PCR primers that yield products diagnostic of wt and disrupted loci. (TIF) [file pone.0152510.s004.tif]

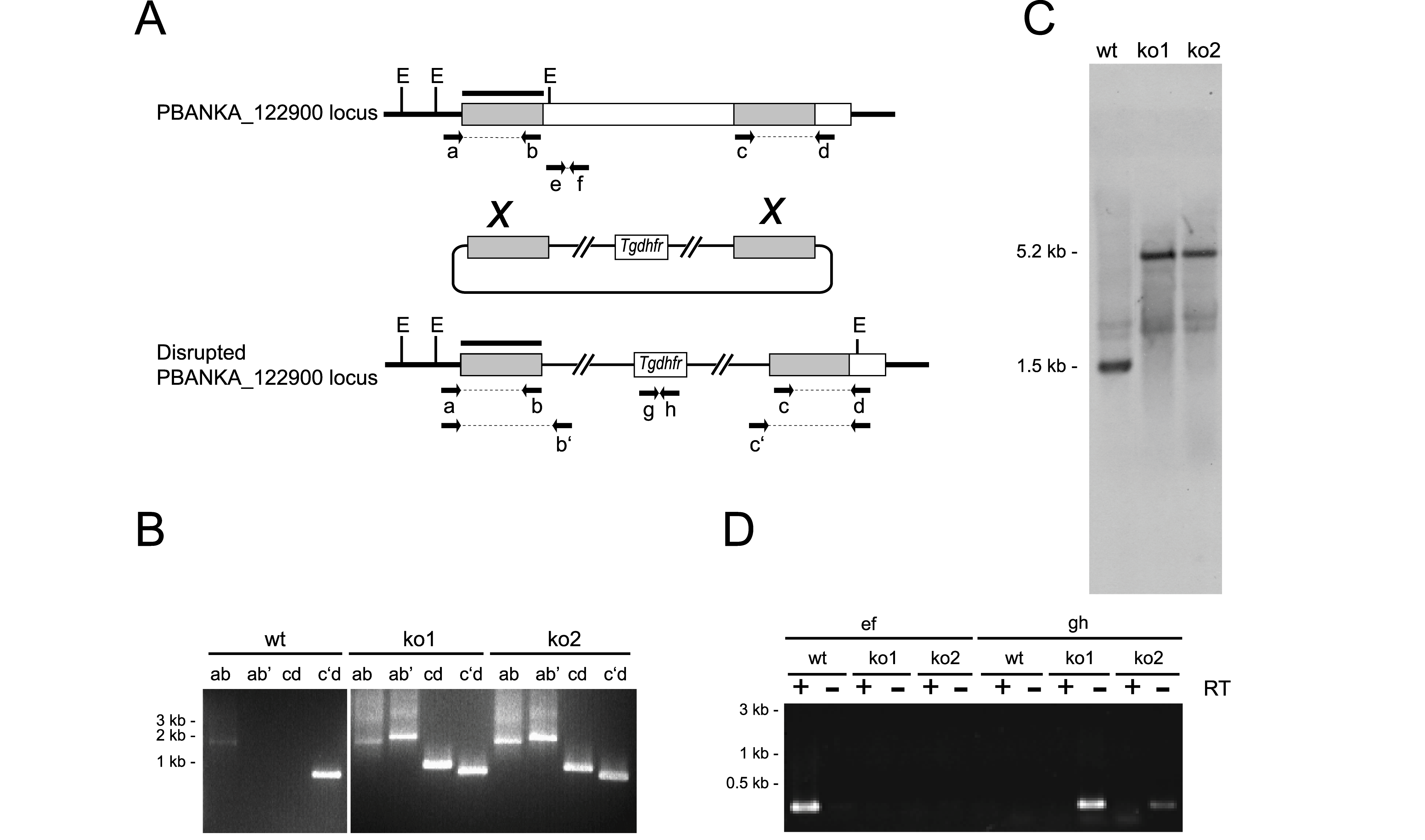

Supplement: S5 Fig — A) Schematic of the PBANKA_122900 locus, disruption plasmid and expected disrupted locus. Thick black lines indicate the 5’- and 3’-UTRs of PBANKA_122900 and boxes indicate the coding sequence; gray boxes indicate the regions of PBANKA_122900 that were used to target homologous recombination within the disruption plasmid; hdhfr indicates the coding sequence of the selectable marker, flanked by thin lines representing the 5’- and 3’-UTRs. Double diagonal lines interrupting the 5’- and 3’-UTRs of the hdhfr cassette indicate that these regions are not in scale. Letters “a” to “d” indicate the locations of PCR primers that yield products diagnostic of wt and disrupted loci; and “e” to “h” indicate the location of primers used in the RT-PCR. The probe used to screen the Southern blot is indicated as a solid horizontal bar above one of the gray boxes. Abbreviations are C, ClaI; and E, EcoRI. B) EtBr-stained agarose gel electrophoresis of PCR products amplified from primer pairs diagnostic of wt locus and integration events. Results from 2 disruptant clones are shown; ko1 and ko2. Different lanes show results with the indicated primer sets which are shown in panel A. C) Southern blot of ClaI and EcoRI digested genomic DNA from wt and disruptant lines showing complete disruption of the PBANKA_122900 gene locus. D) RT-PCR with primer set “ef” shows loss of PBANKA_122900 expression in disruptant parasites. Primer set “gh” shows the presence of the drug selectable marker cassette in transgenic parasites. Reverse transcriptase (RT) and sham controls are indicated above the lanes by “+” and “-”, respectively. The P. berghei gene, ama-1 (PB000821.01.0), was used as a positive control (data not shown). (TIF) [file pone.0152510.s005.tif]

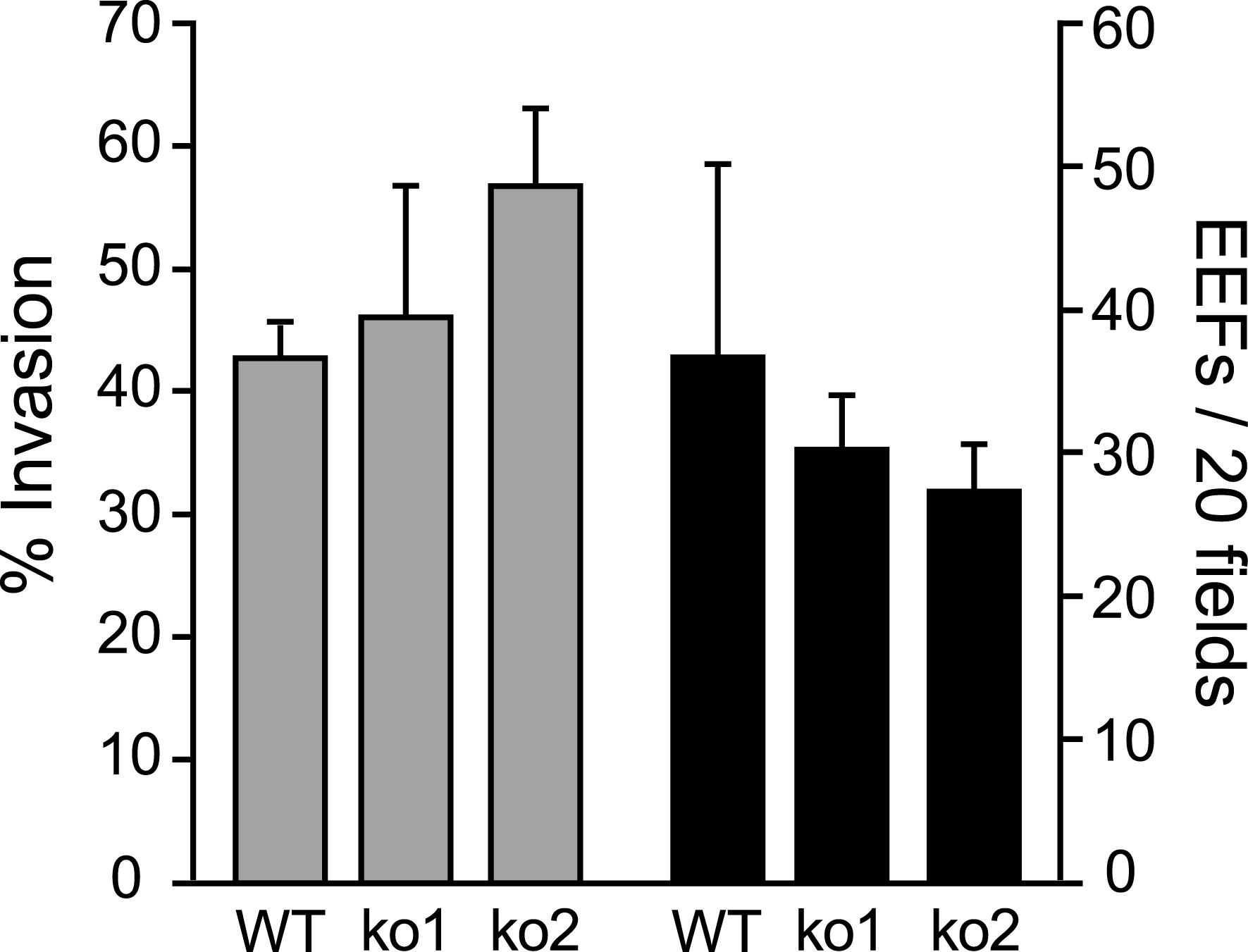

Supplement: S6 Fig — P. berghei wt and 2 clones of PBANKA_122900 KO sporozoites (ko1 and ko2) were added to Hepa 1–6 cells for 1 h and either fixed for invasion assay (grey bars) or grown for an additional 2 days before fixing and staining for exoerythrocytic forms (EEFs, black bars). Fifty and 20 fields per well at 400x magnification were counted for invasion and development assays, respectively. Shown are the means ± SD of triplicates. (TIF) [file pone.0152510.s006.tif]
