# Supplementary material for: The Plasmodium PHIST and RESA-Like Protein Families of Human and Rodent Malaria Parasites
Source: PLoS One. 2016 Mar 29;11(3):e0152510. doi: 10.1371/journal.pone.0152510 (PMC4811531; doi:10.1371/journal.pone.0152510)
Supplement: S1 Table — (DOCX) [file pone.0152510.s007.docx]

| **Target gene** | **Forward primer** | **Reverse primer** |
| --- | --- | --- |
| PF11_0503 | CCCAAAAGAATGTATGAAAA | ATCATTTTTATTTGGTGTGC |
| PF14_0730 | GATGAAGAAGATGATGGCG | TTCACGTCAATCTTAGTTGG |
| PFD1170c | TAACGGATCAAGCTTTTCTA | AACATAGAGAACACCCAACA |
| PFI1770w | AACGGATTCTGTATTAAGCA | TTTCATCACTCCACTGTTTT |
| PFL0050c | ATGGGAAAACAAAGAAAGTT | CAGCATCTAATTCGTTATCAA |
| PFB0105c | GGATATATGAAGGGATCTGG | CATTTTTATTTACATCTCCTGG |
| PFL0060w | TCCCAATTGTAAAACTTCAT | TTTCCATCTAAGGGAGGTAT |
| PFD0090c | GAAGGATATGCTGCTTCAGG | CCCTGGTTATCTTCCTTATTAC |
| MAL7P1_172 | CCTCATATGCAAGAAGAACC | CTTCCTGTCGGTTTTCTAGG |
| PF10_0161 | GCAAATGCTACCAGTAGCAA | CCATCGTTAATTTCATTATTATC |
| PFB0905c | CCCATAAAAGAAACCTTGCTC | GCGTTATTCCTACTTTGAGAACC |
| PFB0920w | GATATGATTCTTCAGATGAACC | GGAATTAACTTCTTCTTCTAAAG |
| PF10_0378 | CAGAAAATGTGTTTGTTGATAAG | ACATTAATTTTGCTTCTTCATCCCC |
| PF11_0512 | CGTATATGGACGTAATCAATATGCG | CCATTTATTGGATAATGTTCTTTGGC |
| PFL0055c | GAGCGTGGTATGAATTATTTATTAAAAG | CGAAATTTCAATTTAGCTATACG |
| PFB0085c | CGTATATCCTACTTCTGAATTGAGTG | CCCTAAATTTTGTTAATGCTTCAGC |
| PF11_0509 | CTATTCAAGAAAATATAGAAGAGG | CCTTCATTAACTGAATATTTTGGTGG |
| PFA0110w | GAAGCAGCTCCAACAATTGAAATCCCCG | GTTGTGGAAAACAGTAGAACC |
| PFE1600w | TGACATGTGGACAAGATTCC | CCATTTCGTGTTTTCCATTC |
| PFE1605w | GAGGGAGAGAAATTTTAATTACG | CATTCCATAAGTCGTGCTCC |
| PFL0900c  *(arginyltRNA synthetase)* | AAGAGATGCATGTTGGTC | GTACCCCAATCACCTACA |
| PBANKA_114540 | GCCGAAGTATGTGACAAGCC | CATTAATTGGTTTCGGATTTCC |
| PBANKA_122900 | CCCTACACGCACATCTTCAG | ATGGTTTCCCATGGATGTTG |
| PBANKA_091440  *(Pbhsp70)* | AGAGAAGCAGCTGAAACAGC | TCCCTTTAATAAATCATGGC |
| PBANKA_091500  *(Pbama-1)* | CAGCCCAAGAAAATATGGG | TTTACAATAACCATCAACCC |
| PBANKA_130070  (*PbCCp1)* | AATAGATAAGCAGGGGGGG | TGTATTCAAATAAATCGATTGG |
| PBANKA_040320  *(PbCSP)* | GTACCATTTTAGTTGTAGCG | CGGGAGCATCGGCAAGTAATC |
