# Supplementary material for: The Plasmodium PHIST and RESA-Like Protein Families of Human and Rodent Malaria Parasites
Source: PLoS One. 2016 Mar 29;11(3):e0152510. doi: 10.1371/journal.pone.0152510 (PMC4811531; doi:10.1371/journal.pone.0152510)
Supplement: S2 Table — (DOCX) [file pone.0152510.s008.docx]

| **Primer name** | **Sequence** |
| --- | --- |
| PFE1600wSXhoI | ctcgagATGAGAATCTTTAATGGATC |
| PFE1600wASBglII | GAagatctTTGATTGTTAGAGACTTCATC |
| PFE1605wSXhoI | ctcgagATGAGGTTTACTAATTCATTAT |
| PFE1605wASBglII | GAagatctTCCAGATTTGTCCTTTGTATTTTC |
| PFE1600wS2 | GAATGGAAAACACGAAATGG |
| PFE1600wAS2 | GCTTGCCTGCATACTCATGTGG |
| PFE1600wmycAS3c | TTCGGCGGCAGATCTTTGATTG |
| PFE1605wS2 | GAGGGAGAGAAATTTTAATTACG |
| PFE1605wAS2 | CATTCCATAAGTCGTGCTCC |
| PFE1605whrpS3c | ATGCAGCTCGAGATGAGG |
| PB848ko5SKpnI | ggtaccCCTGAACAAGAAGTTGAGTATGC |
| PB848ko5ASHindIII and “b” | aagcttCCACATTGGTTCTTCTATTTTACGC |
| PB848ko3SEcoRI and “c” | gaattcGAGGCATTTGATAGATTCG |
| PB848ko3ASBamHI | ggatccGCCGCCGTGCGCATTTCTGG |
| “a” | GCATGATCCCCTCTGATTC |
| “b’ “ | GCTCACCCCTCAAAGCACCAG |
| “c’ “ | CAATGATTCATAAATAGTTGGACTTG |
| “d” | CTGGAATTCTTTCCAAGACTCC |
| “e” PB848rtS5 | GCGTAAAATAGAAGAACCAATGTG |
| “f” PB848rtS5 | CTGAAGATGTGCGTGTAGGG |
| “g” TgDHFRS1 | CCCGAAGAAGCCAGTCGCC |
| “h” TgDHFRAS1 | GGGAAGAGGAAACGACG |
| PB106ko5SHindIII | aagcttCCGCATTGTTCTTGGCGCCG |
| PB106ko5ASPstI | ctgcagCGTGGGTGCGCTATTTTCG |
| PB106ko3SKpnI | ggtaccCACAAAACATAACTCACAATGACG |
| PB106ko3ASEcoRI | gaattcCTTGGCGCATCTTGTTATGAGC |
| 6288 | AACTAGTATGGCTCGTAATTGCGAATG |
| 6289 | AGGATCCCATAGGTTTTGCTCTACAAAATATG |
