# Supplementary material for: The Plasmodium PHIST and RESA-Like Protein Families of Human and Rodent Malaria Parasites
Source: PLoS One. 2016 Mar 29;11(3):e0152510. doi: 10.1371/journal.pone.0152510 (PMC4811531; doi:10.1371/journal.pone.0152510)
Supplement: S3 Table — (DOCX) [file pone.0152510.s009.docx]

| PFB0900c | Revised gene structure, signal peptide and PEXEL identified. |
| --- | --- |
| PFB0905c | Revised gene structure, signal peptide and PEXEL identified. |
| PFE1595c | Revised gene structure, signal peptide and PEXEL identified. |
| PF08_0137 | Revised gene structure, signal peptide and PEXEL identified. |
| PF10_022 | Likely typographical error, should be *phist* gene PF10_0022. Revised gene structure, signal peptide and PEXEL identified. |
| PF10_0161 | Split into two genes, PF10_0161 and PF10_0161a, each with a single PHIST domain and signal peptides/PEXEL motifs. |
| PF10_0162 | Revised gene structure, signal peptide and PEXEL identified. |
| PF10_0503 | Not in PlasmoDB. Typo? Should be *phist* gene PF11_0503? |
| PFB0080c | PHIST domain present. |
| PFE1180w | Likely typographical error, should be *phist* gene PFD1180w. |
| MALP1.19 | Revised name, PFF0075c. Revised gene structure, signal peptide and PEXEL identified. |
| MAL8P1.2 | Revised gene structure, signal peptide and PEXEL identified. |
| PFI1790w | Revised gene structure, signal peptide and PEXEL identified. |
| PF14_0731 | Revised to be *phist* gene PF14_0730. Revised gene structure, signal peptide and PEXEL identified. |
| PF14_0746 | Revised gene structure, signal peptide and PEXEL identified. |
| PFA0100c | Likely pseudogene. |
| PFD1210w | Likely pseudogene. |
| PF10_0014 | Not found in PlasmoDB. |
| PF11_0012 | Pseudogene, PFEMP1 fragment. |
| PF11_0514 | Likely pseudogene. |
| MAL13P1.59 | Revised gene structure, signal peptide and PEXEL identified. |
| PF14_0748 | Revised gene structure, signal peptide identified, variant or absent PEXEL. |
| **Additional *phist* genes identified in the present study** | |
| PFA0735w | Almost identical to *phist* gene MAL8P1.163. |
| PFD1180w | See note above regarding PFE1180w. |
| MAL7P1.224 |  |
| MAL7P1.225-a | Alternative gene model in PlasmoDB, MAL7P1.225-b. |
| PF10_0161a | Seen note above for PF10_0161. |
| PF11_0503 | See note for PF10_0503 above. |
| PFL0060w | Coding region of gene possibly truncated at 3’ end. |
| PFL2590w | Almost identical to *phist* gene PF14_0763. |
| PF14_0744 | Described as gametocyte-expressed gene in Eksi et al. (2005). |
| PF14_0745 | Described as gametocyte-expressed gene in Eksi et al. (2005). |
| **Predicted *phist* pseudogenes** | |
| MAL7P1.220 | Three exon gene structure. |
| PFA0100c | Four exon gene structure. |
| MAL8P1.215 | Three exon gene structure. |
| PF10_0007 | Three exon gene structure. |
| PFL2650w | Predicted gene fragment. |
| MAL13P1.525 | Predicted gene fragment. |
| PFF0085w | Stop codon within ORF. |
| PFL2595w | Three exon gene structure. |
| PF11_0514 | Predicted gene fragment. |
| PFD1210w | Predicted gene fragment. |
| MAL13P1.11 | Stop codon within ORF. |
| MAL13P1.58 | Predicted gene fragment. |
