# Supplementary material for: The Plasmodium PHIST and RESA-Like Protein Families of Human and Rodent Malaria Parasites
Source: PLoS One. 2016 Mar 29;11(3):e0152510. doi: 10.1371/journal.pone.0152510 (PMC4811531; doi:10.1371/journal.pone.0152510)
Supplement: S4 Table — (DOCX) [file pone.0152510.s010.docx]

|  |  | **Oocyst** | **number** |  |
| --- | --- | --- | --- | --- |
| **Experiment**  **number** | **Parasite population** | **Mean** | **Range** | **Prevalence (%) (n=20)** |
| **1** | wt | 98 | 0-378 | 90 |
|  | ko 1 | 93 | 0-358 | 75 |
|  | ko 2 | 222 | 0-572 | 90 |
| **2** | wt | 49 | 0-209 | 95 |
|  | ko 1 | 19 | 0-77 | 85 |
|  | ko 2 | 51 | 0-173 | 70 |
