# Supplementary material for: The Plasmodium PHIST and RESA-Like Protein Families of Human and Rodent Malaria Parasites
Source: PLoS One. 2016 Mar 29;11(3):e0152510. doi: 10.1371/journal.pone.0152510 (PMC4811531; doi:10.1371/journal.pone.0152510)
Supplement: S5 Table — (DOCX) [file pone.0152510.s011.docx]

| **Experiment**  **number** | **Parasite population** | **Midgut sporozoites per mosquito** | **Salivary gland sporozoites per mosquito** | | |
| --- | --- | --- | --- | --- | --- |
|  |  | **Day 14** | **Day 17** | **Day 19** | **Day 21** |
| **1** | wt | 3,200 | 2,200 | - | 18,600 |
|  | ko 1 | 20,000 | 7,000 | - | 14,600 |
|  | ko 2 | 2,800 | 1,100 | - | 18,200 |
| **2** | wt | 5,000 | - | 7,700 | - |
|  | ko 1 | 2,500 | - | 5,700 | - |
|  | ko 2 | 2,600 | - | 18,800 | - |
