# Supplementary material for: The Plasmodium PHIST and RESA-Like Protein Families of Human and Rodent Malaria Parasites
Source: PLoS One. 2016 Mar 29;11(3):e0152510. doi: 10.1371/journal.pone.0152510 (PMC4811531; doi:10.1371/journal.pone.0152510)
Supplement: S6 Table — (DOCX) [file pone.0152510.s012.docx]

| **Experiment and mouse strain** | **Parasite line** | **Number of sporozoites injected^a^** | **Number of mice positive/number of mice injected^b^** | **Prepatent period**  **(day)^c^** |
| --- | --- | --- | --- | --- |
| **Experiment 1** |  |  |  |  |
| Swiss Webster | wt | 1,000 | 5/5 | 4 |
|  | wt | 100 | 3/5 | 5 |
|  | ko1 | 1,000 | 5/5 | 4 |
|  | ko1 | 100 | 2/5 | 4 |
| **Experiment 2** |  |  |  |  |
| Swiss Webster | wt | 1,000 | 4/5 | 5 |
|  | ko1 | 1,000 | 4/5 | 5 |
|  | ko2 | 1,000 | 4/5 | 5 |

**a**. Number of salivary gland sporozoites inoculated by tail IV injection.

**b**. Number of mice with blood parasitemia /number of mice inoculated.

**c.** Number of days from inoculation until detection of blood parasitemia.
